# Sex-specific patterns of symptom burden, psychological distress, and work productivity impairment in post-acute sequelae of COVID-19: a 10-month dynamic cohort study

**DOI:** 10.1186/s12890-026-04741-x

**Published:** 2026-09-19

**Authors:** Andrea Sauer, Lennart Boblitz, Nora Drick, Miriam Wiestler, Michèle Scharbau, Philipp Pries, Benjamin-Alexander Bollmann, Marius M. Hoeper, Isabell Pink

**Affiliations:** 1 Department of Respiratory Medicine and Infectious Diseases, Hannover Medical School, Carl-Neuberg-Straße 1, Hannover, Germany; 2 Member of the German Centre for Lung Research (DZL), Biomedical Research in End Stage and Obstructive Lung Disease Hanover (BREATH), Hannover, Germany; 3 Department of General Internal Medicine, Clinics of the City of Cologne, Cologne, Germany; 4 Department of Gastroenterology, Hepatology, Infectious Diseases and Endocrinology, Hannover Medical School, Hannover, Germany; 5 Department of Internal Medicine, Vinzenz Hospital Hannover, Hannover, Germany; 6 Department of Internal Medicine, Zurich Rehabilitation Centres, Davos Clavadel Clinic, Davos, Switzerland

**Keywords:** COVID-19, PASC, Sex-specific, Work produtivity impairment

## Abstract

**Background:**

A considerable proportion of patients experience post-acute sequelae of COVID-19 (PASC). Increasing evidence suggests sex-related differences, yet systematic longitudinal analyses remain limited. This study aimed to examine sex-specific differences in symptoms, psychological burden, work productivity and physical activity over ten months following acute COVID-19.

**Methods:**

In this prospective, dynamically enrolled cohort study, patients with ongoing symptoms after confirmed SARS-CoV-2 infection were followed over a 10-month observation period. Patients conducted at the post-COVID outpatient clinic of the department of respiratory medicine and infectious diseases at Hannover Medical School, Germany. The assessment included patient-reported outcome measures, pulmonary function tests, six-minute walk tests, and capillary blood gas analysis. All outcome comparisons between sexes were adjusted for age, BMI, COVID-19 severity, and comorbidities using generalised linear mixed-effects models, with sex-by-time interaction terms tested to assess whether differences changed over follow-up, and p-values corrected for multiple testing across all outcome comparisons using the Holm method. A sensitivity analysis was performed in the subgroup of patients with complete follow-up at all four visits.

**Results:**

Five hundred ninety patients attended to our clinic and a total of 433 patients were eligible and consecutively enrolled. Males were older (56 years vs. 52 years, *p =* 0.002), had more cardiovascular comorbidities (16% vs. 5%, *p <* 0.001), and experienced more severe COVID-19 (28% vs. 8%, *p <* 0.001). After adjustment for age, BMI, COVID-19 severity, and comorbidities, and Holm correction for multiple testing across all 27 outcome comparisons, females showed higher fatigue (Fatigue Assessment Scale, adjusted β = 3.3, Holm-adjusted *p =* 0.006), higher anxiety (GAD-2, β = 0.5, *p =* 0.021), lower quality of life (EQ-5D-3L, β = -0.6, *p =* 0.004), and greater work productivity impairment, both as a continuous score (WPAI-VAS, β = 1.3, *p =* 0.031) and as a binary indicator of clinically relevant impairment (OR = 3.0, *p =* 0.020). Females also had higher percent-predicted (pp) total lung capacity (*p <* 0.0001) and lower pCO_2_ (*p <* 0.0001), whereas males had higher pp diffusion capacity (*p =* 0.001). Headache (*p <* 0.001) and chest tightness (*p =* 0.011) were reported more frequently by females. None of these differences varied significantly over the 10-month follow-up (all sex-by-time interaction *p ≥* 0.41), indicating no evidence that these differences changed over time. No significant sex differences remained after adjustment for depression (PHQ-2), lung function parameters, sex/height-adjusted exercise capacity, post-exercise dyspnoea or oxygen saturation, or most acute-COVID-19 symptoms other than headache and chest tightness. Regression models separately for each sex revealed fever (OR = 2.48, 95% CI: 1.11–5.57, *p =* 0.027) and diarrhoea (OR 2.33, 95%CI: 1.05–5.16, *p =* 0.038) during acute COVID-19 were associated with higher odds of work productivity impairment (WPAI-VAS ≥ 2) in males. In females, higher BMI (OR = 2.06, 95% CI: 1.13–3.76, *p =* 0.018) and dyspnoea during acute COVID-19 (OR = 4.38, 95% CI: 1.55–12.40, *p =* 0.005) were associated with increased odds. Formal interaction analyses showed a significant interaction only for dyspnoea after Holm correction.

**Conclusions:**

After accounting for baseline imbalance in age, BMI, COVID-19 severity, and comorbidities, and after correcting for multiple testing, sex-specific differences persisted for fatigue, anxiety, quality of life, and work productivity impairment, as well as selected lung function parameters. These differences did not change significantly across the 10-month observation period and did not show a significant sex-by-time interaction. The findings support the need for individualised, sex-specific follow-up focused on fatigue, psychological burden, and work productivity in patients with PASC.

**Supplementary Information:**

The online version contains supplementary material available at https://doi.org/10.1186/s12890-026-04741-x.

## Introduction

Coronavirus disease 2019 (COVID-19), caused by the severe acute respiratory syndrome coronavirus 2 (SARS-CoV-2), has had profound global health consequences, affecting more than 700 million individuals and resulting in over seven million deaths worldwide as of 2024 [1]. While the acute phase is often self-limiting, approximately 10–15% of patients develop persistent symptoms extending beyond the resolution of the initial infection, referred to as post-acute sequelae of COVID (PASC) [2, 3]. The World Health Organisation (WHO) defines PASC as a constellation of symptoms that usually occur within three months of COVID-19 onset, last for at least two months, and cannot be explained by an alternative diagnosis [4].

PASC can occur irrespective of initial disease severity and encompasses a broad range of manifestations, including fatigue, dyspnoea, impaired pulmonary function, reduced exercise capacity, and psychological symptoms such as depression and anxiety [5, 6]. Although the underlying pathophysiology remains incompletely understood, emerging evidence points to persistent immune dysregulation, endothelial dysfunction, and autonomic imbalance as potential causes [7].

Several studies report that females are disproportionately affected by PASC, identifying female sex as an independent risk factor, whereas in acute COVID-19, males show a higher risk of severe acute disease and mortality [1, 8]. As is known, sex is an established biological factor that affects susceptibility to infectious diseases, immune responses, and recovery trajectories [9].

Recent longitudinal studies suggest sex-specific differences in pulmonary function, persistence of symptoms, physical performance and rehabilitation outcomes among patients with PASC [5, 10, 11]. A systematic review and meta-analysis by Sylvester et al. further identified sex-specific disparities in symptom burden. Females exhibit greater burden in fatigue, psychological distress, and functional impairment, whereas males demonstrate more pronounced objective impairments in lung function and exercise capacity, although findings remain heterogeneous [9, 12]. The observed sex-specific distinctions in PASC may be influenced by multiple factors, including differences in immune regulation, hormonal influences, endothelial dysregulation, and gender-related social and occupational exposures [13]. Moreover, Gebhard et al. described role- and gender-specific stressors, such as care work and financial burden, as potential modifying factors [9]. However, no cause has been identified with certainty until now.

Importantly, PASC has been shown to substantially reduce work ability, functional independence and therefore social participation [5, 11]. Kautzky et al. identified depressive and anxiety symptoms as potential contributors to quality of life and work ability in individuals with PASC. However, research using validated work ability and productivity assessments remains limited, and very few studies have investigated whether such functional impairment differs between females and males.

Despite growing evidence that biological sex affects multiple dimensions of PASC, studies comparing females and males across clinically relevant domains, using standardised outcome measures and follow-up periods exceeding three months, remain scarce. As PASC is a novel, dynamic, fluctuating and prolonged condition with heterogeneous clinical manifestations, repeated assessments across a follow-up period may provide additional insight into sex-specific patterns of symptoms and functional impairment. This prospective, dynamically enrolled cohort study investigates sex-specific risk profiles in patients with PASC associated with work productivity impairment. The primary aim was to describe symptoms, psychological burden, lung function, physical performance, and work productivity impairment between female and male patients over a 10-month observation period, using models that account for the unbalanced, dynamic structure of the cohort. In addition, the study aimed to identify factors associated with work productivity impairment separately in female and male participants, thereby characterising sex-specific risk profiles. The study does not assess differences in the incidence of PASC but focuses exclusively on outcomes among patients with established PASC.

## Methods

### Study design, patient characteristics and symptoms

**Fig. 1:**
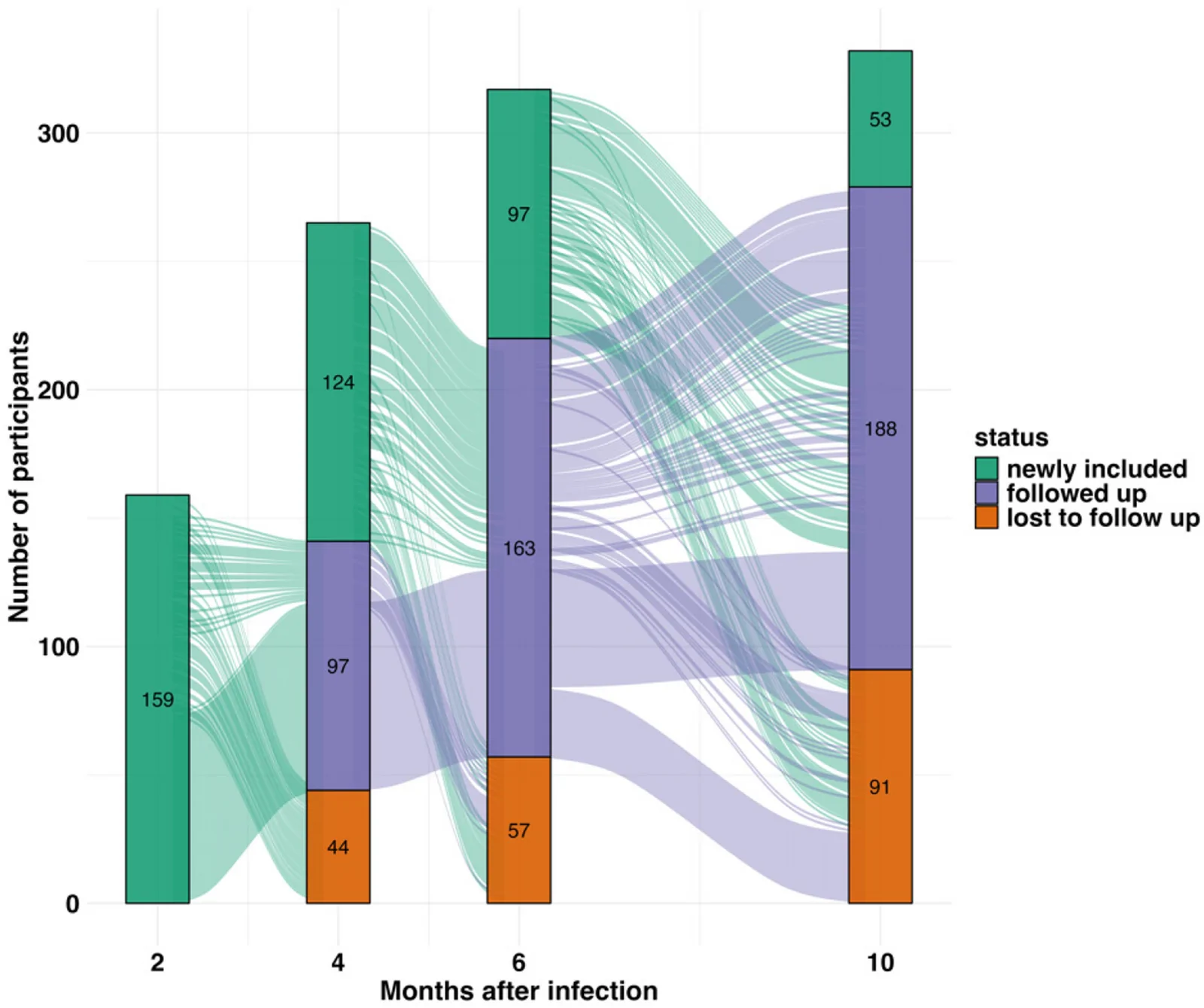
Patients' Alluvial Plot with participants dynamic flow and follow-up status across study visits (2-10 months after infection)

This prospective cohort study was performed at the post-COVID outpatient clinic of the Department of Respiratory Medicine and Infectious Diseases at Hannover Medical School, Germany. Patients were prospectively recruited between May 2020 and March 2024 following a SARS-CoV-2 infection, as detected by a virus-specific Polymerase Chain Reaction (PCR) test or by the presence of specific antibodies against the SARS-CoV-2 nucleocapsid (N). All patients complained about new-onset, ongoing symptoms after COVID-19. During the recruitment period, patients were recruited consecutively from the outpatient clinic and no eligible patient declined participation. Of 590 patients attending to the outpatient clinic, 433 were eligible and included in the study. Repeated measures were aligned to time since SARS-CoV-2 infection (2, 4, 6 and 10 months after infection). Due to the outpatient nature of recruitment, participants entered the study at different time points, and not all participants contributed data at every assessment point. Sex was assessed as documented in the medical records; no assessment of the gender identity was performed. The course of acute COVID-19 was classified into four severity levels: asymptomatic, mild (outpatient), moderate (inpatient on a regular ward), and severe (inpatient requiring treatment in the intensive care unit, ICU). Figure 1 shows the flow of the dynamic patient cohort during the study period, visualising the different attendance of patients. The participants were present in various ways, with some attending only individual visits, others only in the early stages, and yet others only at late visits. At their first visit patients were asked about comorbidities and their symptoms during the acute phase of the disease, as shown in Table 1. Symptoms asked for during the acute phase were dyspnoea, cough, headache, chest tightness/pain, fever, diarrhoea, loss of smell and fatigue. For symptoms persisting in the post-acute phase, we asked about chest tightness, arthritis, fever, cough, headache, reflux, sickness, sputum production, syncope and weight loss. Symptoms were assessed after the infection and patient-reported outcome measures (PROMs) were completed at each visit, as well as lung function and physical activity measurements. Education level, classified according to International Standard Classification of Education (ISCED 2011), was also assessed at baseline.

**Table 1:** Patients' characteristics and symptoms of acute COVID-19

	Male (*N* = 196)	Female (*N* = 237)	Total (*N* = 433)	Holm adjusted *P* value
Age (years)				0.026^a^
Median (Q1, Q3)	56 (44, 65)	52 (40, 59)	53 (41, 62)	
Missing	0	0	0	
COVID-19 intensity				< 0.001^b^
Asymptomatic	0 (0%)	2 (1%)	2 (0%)	
Mild	98 (50%)	193 (82%)	291 (68%)	
Moderate	43 (22%)	22 (9%)	65 (15%)	
Severe/ICU	54 (28%)	18 (8%)	72 (17%)	
Missing	1	2	3	
BMI (kg/m^2^)				< 0.001^a^
Median (Q1, Q3)	28 (24, 31)	25 (22, 29)	26 (23, 30)	
Missing	0	0	0	
Education ISCED Level				0.055
Primary education	6 (3%)	7 (3%)	13 (3%)	
Lower secondary education	18 (10%)	4 (2%)	22 (6%)	
Upper secondary education	57 (32%)	72 (34%)	129 (33%)	
Post-secondary non-tertiary education	20 (11%)	42 (20%)	62 (16%)	
Bachelor’s or equivalent	43 (24%)	40 (19%)	83 (22%)	
Master’s or equivalent	20 (11%)	34 (16%)	54 (14%)	
Doctoral or equivalent	12 (7%)	11 (5%)	23 (6%)	
Missing	20	27	47	
Employment status*				0.066^b^
Full-time employment	47 (56%)	23 (35%)	70 (47%)	
Part-time employment	9 (11%)	19 (29%)	28 (19%)	
Not employed	25 (30%)	17 (26%)	42 (28%)	
Other	3 (4%)	7 (11%)	10 (7%)	
Missing	112 (57%)	171 (72%)	283 (65%)	
Obesity (BMI ≥ 30 kg/m^2^)				0.310^b^
No	136 (69%)	186 (78%)	322 (74%)	
Yes	60 (31%)	51 (22%)	111 (26%)	
Missing	0	0	0	
Cardiovascular diseases				0.008^b^
No	165 (84%)	224 (95%)	389 (90%)	
Yes	31 (16%)	13 (5%)	44 (10%)	
Missing	0	0	0	
Depression				1.000^b^
No	184 (94%)	216 (91%)	400 (92%)	
Yes	12 (6%)	21 (9%)	33 (8%)	
Missing	0	0	0	
Diabetes mellitus				0.066^b^
No	171 (87%)	225 (95%)	396 (91%)	
Yes	25 (13%)	12 (5%)	37 (9%)	
Missing	0	0	0	
Arterial hypertension				< 0.001^b^
No	132 (67%)	206 (87%)	338 (78%)	
Yes	64 (33%)	31 (13%)	95 (22%)	
Missing	0	0	0	
Chronic liver disease				0.163^b^
No	187 (95%)	235 (99%)	422 (97%)	
Yes	9 (5%)	2 (1%)	11 (3%)	
Missing	0	0	0	
Chronic renal insufficiency				0.904^b^
No	185 (94%)	231 (97%)	416 (96%)	
Yes	11 (6%)	6 (3%)	17 (4%)	
Missing	0	0	0	
Reflux disease				1.000^b^
No	193 (98%)	232 (98%)	425 (98%)	
Yes	3 (2%)	5 (2%)	8 (2%)	
Missing	0	0	0	
Obstructive lung diseases				1.000^b^
No	172 (88%)	204 (86%)	376 (87%)	
Yes	24 (12%)	33 (14%)	57 (13%)	
Missing	0	0	0	
Restrictive lung diseases				1.000^b^
No	192 (98%)	235 (99%)	427 (99%)	
Yes	4 (2%)	2 (1%)	6 (1%)	
Missing	0	0	0	
Smoking status				0.122^b^
Current	11 (6%)	9 (4%)	20 (5%)	
Former	91 (47%)	79 (34%)	170 (40%)	
Never	93 (48%)	146 (62%)	239 (56%)	
Missing	1	3	4	
Symptoms of acute COVID-19				
Dyspnoea				1.000^b^
No	68 (35%)	97 (41%)	165 (38%)	
Yes	125 (65%)	139 (59%)	264 (62%)	
Missing	3	1	4	
Cough				0.163^b^
No	80 (41%)	71 (30%)	151 (35%)	
Yes	113 (59%)	165 (70%)	278 (65%)	
Missing	3	1	4	
Headache				< 0.001^b^
No	76 (39%)	46 (19%)	122 (28%)	
Yes	117 (61%)	190 (81%)	307 (72%)	
Missing	3	1	4	
Chest tightness				0.991^b^
No	106 (55%)	112 (47%)	218 (51%)	
Yes	87 (45%)	124 (53%)	211 (49%)	
Missing	3	1	4	
Fever				0.991^b^
No	61 (32%)	91 (39%)	152 (35%)	
Yes	132 (68%)	145 (61%)	277 (65%)	
Missing	3	1	4	
Diarrhoea				1.000^b^
No	141 (73%)	177 (75%)	318 (74%)	
Yes	52 (27%)	59 (25%)	111 (26%)	
Missing	3	1	4	
Loss of smell				0.006^b^
No	119 (62%)	104 (44%)	223 (52%)	
Yes	74 (38%)	132 (56%)	206 (48%)	
Missing	3	1	4	
Fatigue				0.002^b^
No	47 (24%)	24 (10%)	71 (17%)	
Yes	146 (76%)	212 (90%)	358 (83%)	
Missing	3	1	4	
SARS-CoV-2 vaccinations status at first visit				0.041^b^
No vaccination	107 (55%)	94 (40%)	201 (46%)	
One vaccination	48 (24%)	56 (24%)	104 (24%)	
Two vaccinations	19 (10%)	42 (18%)	61 (14%)	
Three or more vaccinations	22 (11%)	45 (19%)	67 (15%)	

*Abbreviation*: *BMI* Body mass index, *ISCED* International Standard Classification of Education, *SARS-CoV-2* Severe acute respiratory syndrome coronavirus 2

^a^Wilcoxon rank sum test

^b^Pearson’s Chi-squared test

^*^Employment status was not a mandatory field and was therefore not completed by all participants, resulting in substantial missingness; reported percentages are calculated among participants with avalaible data only

This design yields repeated cross-sectional assessments within a dynamically enrolled cohort rather than complete individual trajectories for most patients; only 46 of 433 patients (10.6%) contributed data at all four visits. We therefore avoid interpreting the follow-up data as tracked within-patient trajectories except where explicitly noted (sensitivity analysis, complete-case subgroup). Sex-specific attrition was formally assessed (see Statistical analysis) to evaluate whether the composition of the cohort at each visit differed systematically between females and males; this differed significantly at the 4-month visit (Holm-adjusted *p =* 0.045) but not at 2, 6, or 10 months (Holm-adjusted *p =* 0.204–0.256).

The study was conducted in accordance with the Declaration of Helsinki, and informed consent was obtained from every patient. This study was approved by the institutional review board at Hannover Medical School (#9226_BO_K_2020).

### Psychological burden and work ability

Various PROMs were used to investigate psychological stress. To assess Quality of life (QoL), depression and anxiety, we used the Generalised Anxiety Disorder – 2 item Score (GAD-2), Patient Health Questionnaire-2 (PHQ-2) and Quality of Life – European Quality of Life 5 Dimensions 3 Level Version questionnaire (EQ-5D-3L), as these are validated standard questionnaires [14–16]. The GAD-2 score assesses the frequency of core anxiety symptoms—nervousness and uncontrollable worry—over the past two weeks. Each item is rated on a scale from 0 to 3; a score > 3 indicates a positive screening for present anxiety symptoms [17]. Furthermore, we used the PHQ-2 Score to identify depressive symptoms. It assesses the frequency of depressive moods and anhedonia over the past two weeks, rated on a 4-point Likert scale from 0 (no symptoms) to 3 (everyday symptoms) [18]. The EQ-5D-3L Score is an instrument used to measure health-related quality of life. It comprises five dimensions – mobility, self-care, usual activities, pain/discomfort, and anxiety/depression—rated on three levels: no problems (scale 1), some problems (scale 2), and extreme problems (scale 3). The answers to the questionnaire are converted into an index value using standardised calculation methods. It results in a five-digit health state code that represents the individual’s self-reported health [19]. The Fatigue Assessment Scale (© FAS Fatigue Assessment Scale: ild care foundation www.ildca re.nl) was used to assess fatigue levels (no fatigue, fatigue and extreme fatigue) [20]. The scale ranges from 10 to 50, with a score of < 22 indicating no fatigue, scores between 22 and 34 reflecting the presence of fatigue and scores ≥ 35 indicating extreme fatigue. Moreover, we assessed limitations in daily life due to PASC using a self-administered questionnaire based on the post-COVID-19 functional status scale (PCFS) [21]. This questionnaire utilizes a five-point Likert-scale to address the following domains: COVID-19-specific symptoms (symptoms state), pain or psychological burden (psychiatric state), and daily physical performance (daily routine state) and general limitations (common state). Results were categorized into three distinct groups: improved, stable, and worsened. This questionnaire was developed by our group for use in this cohort; the full instrument is provided in the supplementary material of the original publication describing its development and use [22].

To further investigate the impact on work in participants who reported being in paid employment at the respective study visit. Employment status was assessed at each individual visit. We employed item 5 of the Work Productivity and Activity Impairment – General Health (WPAI-GH) questionnaire. This item contains of a visual analogue scale (VAS, WPAI-VAS) ranging from 0 (work is not affected) to 10 (complete limitation of working) and focusses on the productivity impairment due to health problems [23]. There is no established cut-off value for the WPAI or the VAS defining a relevant impairment. We have based our assessment on the minimal clinically important difference (MCID) of the WPAI-GH (items 1–6) of 20% [24]. Therefore, the geometric means of the WPAI-VAS were dichotomized at a cut-off of ≥ 2 to define clinically relevant impairment. We have assumed that a score of ≥ 2, that is, 20% or higher compared to the absence of limitation, can be considered to constitute a relevant restriction.

### Physical activity and pulmonary function test

In addition to an exercise test to assess patients’ physical capacity, we also performed pulmonary function tests (PFT) at each visit. If patients were able to perform the physical activity, a 6-min walking test was accomplished at every visit. The distance (6MWD) expressed as an absolute value and adjusted for age, sex, height and weight (= percent predicted, pp6MWD) was assessed with continuous measurement of peripheral oxygen saturation (SpO2) and heart rate by pulse oximetry [25]. Furthermore, dyspnoea was queried before and after the test using the ten points BORG Scale (0 = Nothing at all, 10 = Very, very strong) [26]. For PFT, a body plethysmography was performed and percent predicted forced expiratory volume in one second (ppFEV_1_), percent predicted forced expiratory vital capacity (ppFVC_ex_), percent predicted total lung capacity (ppTLC), as well as percent predicted diffusion capacity for carbon monoxide (ppDLCO) were assessed. Blood gases from arterialized capillary blood were measured at rest without oxygen supplementation, and values for partial pressure of oxygen (pO_2_) and carbon dioxide (pCO_2_) were documented.

### Statistical analysis

Normal distribution was assessed using the Shapiro–Wilk test. For categorical variables, absolute frequencies were calculated. For continuous variables, the median and interquartile range (IQR; 25th and 75th percentiles) were reported. Group differences were evaluated using the Wilcoxon rank sum test, for continuous variables, as they were non-normally distributed, and Pearson's Chi-squared test for categorical variables. To account for multiple testing within group comparisons and post hoc analyses of baseline characteristics, the Holm method was used to adjust p-values. A two-sided significance level of 5% was applied throughout.

A comparative analysis of baseline characteristics was conducted to examine differences between female and male participants using the tests described above, with Holm correction for multiple testing (Table 1). Post hoc cellwise comparison was performed using standardised residuals, with a cut-off value of 2, indicating significant over- (≥ 2) or underrepresentation (≤ −2) relative to expected frequencies. Because patients were recruited and followed dynamically, with unequal numbers of assessments per patient and differing time of cohort entry, all outcome comparisons across the follow-up period were modelled using generalised linear mixed-effects models (GLMMs; lme4 package [27]) with a patient-specific random intercept, which adjusts unbalanced repeated measurements. Continuous days since infection was used to reflect the dynamic, individually timed follow-up structure. For every outcome reported in the Results we fitted an unadjusted model with sex, time, and their interaction, and an adjusted model additionally including age, BMI, COVID-19 severity, and comorbidities (cardiovascular disease, diabetes, hypertension) as fixed effects. The sex-by-time interaction term was used to formally test whether sex differences changed across the follow-up period; p-values for these interaction terms, and separately for the adjusted sex main-effect terms, were corrected for multiple testing across all outcomes using the Holm method. To assess whether differential attrition by sex affected these estimates, we formally compared the distribution of number-of-visits-attended and newly included/followed up/lost to follow up status between sexes at each visit window (Chi-squared/Fisher's exact test, Holm-corrected across visits), and performed a sensitivity analysis restricting the adjusted models to the subgroup of patients with complete data at all four visits (*n* = 46). Odds ratios (ORs) and regression coefficients with 95% confidence intervals (95% CI) were derived from the GLMMs. Results were visualised using forest plots on a logarithmic scale (ggplot2 package [28]). All statistical analyses and figures were generated using R Studio (Version 4.5.1 (2025–06–13), Posit Software, Boston, MA, USA).

## Results

### Patient characteristics and symptoms

Table 1 presents the baseline characteristics of the cohort. In total, 433 patients were recruited between May 2020 and March 2024 as part of a dynamic patient cohort. Figure 1 illustrates the flow of patients during the study period. 172 participants attended to one appointment, 120 to two, 95 to three and 46 to all four appointments. Further details of female and male participants newly included, followed up or lost to follow up are listed in Supplementary Table 1. Of this cohort, 237 (55%) participants were female and 196 (45%) were male according to their sex, with no inquiry into gender identity. Males were found to be significantly older and to have experienced a more severe course of the acute COVID-19 (moderate and severe). Furthermore, male patients exhibited higher BMI, as well as greater prevalence of comorbidities such as cardiovascular disease and arterial hypertension. Regarding the manifestation of acute symptoms, female subjects reported a higher prevalence of headache, loss of smell, and fatigue. There was a significant difference in vaccination status, and post-hoc analyses of the standardised residuals (std. res) showed that male participants were significantly more likely to be unvaccinated (std. res = + 3.10) and less likely to have two vaccinations (std. res = −2.39) or ≥ 3 vaccinations (−2.22). Females showed the opposite pattern.

### Formal, adjusted comparison of outcomes between sexes

To address baseline imbalance between sexes (Table 1) and the possibility that follow-up differed structurally between males and females, all 27 outcome comparisons reported above were re-analysed using generalised linear mixed-effects models with a patient-specific random intercept, adjusted for age, BMI, COVID-19 severity, and comorbidities (cardiovascular disease, diabetes, hypertension), with sex-by-time interaction terms tested to assess whether differences changed over the follow-up period. P-values were corrected for multiple testing across all 27 comparisons using the Holm method. After this adjustment and correction, 11 of 27 outcomes remained statistically significant (see Table 2): fatigue (FAS), anxiety (GAD-2), quality of life (EQ-5D-3L), work productivity impairment (WPAI-VAS, both continuous and dichotomised at WPAI ≥ 2), ppTLC, ppDLCO, pCO2, headache, and chest tightness. Absolute 6MWD in meters also remained significant, but the adjusted pp6MWD did not, suggesting the absolute-distance difference primarily reflects anthropometric differences rather than a PASC-specific functional impairment. No significant sex difference remained after adjustment for depressive symptoms (PHQ-2), spirometric parameters (ppFEV1, ppFVCex), post-exercise dyspnoea (BORG) or oxygen saturation, pO2, or acute-COVID-19 symptoms other than headache and chest tightness. None of the sex-by-time interaction terms reached significance after Holm correction (all *p ≥* 0.41), indicating that, where present, sex differences were consistent across the follow-up period of ten months rather than emerging or resolving over time.

**Table 2:** Comparison of all 27 clinical, psychological, and functional outcomes between female and male participants, before and after adjustment for age, BMI, COVID-19 severity, and comorbidities, with Holm correction for multiple testing (*n* = 433)

Outcome	*P* value	Holm adjusted *p* value
6MWD (m)	0.0000	0.0000
pCO_2_	0.0000	0.0000
ppTLC	0.0000	0.0000
Headache	0.0000	0.0003
ppDLCO	0.0000	0.0011
EQ-5D-3L Score	0.0002	0.0044
FAS Score	0.0003	0.0057
Chest tightness	0.0005	0.0108
Dichotomised WPAI (≥ 2)	0.0011	0.0204
GAD-2 score	0.0012	0.0212
WPAI Score	0.0018	0.0305
ppFEV_ex_	0.0089	0.1425
Nightsweat	0.0108	0.1626
Cough	0.0121	0.1696
pp6MWD	0.0199	0.2583
BORG post 6MWT	0.0200	0.2583
PHQ-2 Score	0.0499	0.5492
ppFEV_1_	0.6004	1.0000
SpO_2_ post 6MWT	0.1751	1.0000
PO_2_	0.2728	1.0000
Arthritis	0.8344	1.0000
Fever	0.9237	1.0000
Reflux	0.6057	1.0000
Sickness	0.5524	1.0000
Sputum	0.6184	1.0000
Syncope	0.9895	1.0000
Weight loss	0.5973	1.0000

*Abbreviations*: *FAS* Fatigue Assessment Scale, *GAD-2* Generalized anxiety disorder 2- item, *PHQ2* Patient Health Questionnaire—2, *EQ-5D-3L* European quality of life – 5 dimensions 3 Level Version Questionnaire, *WPAI-VAS *Work Productivity and Ability Index – Visual Analog Scale, *6MWD (m)* six-minute walking distance (meters), *pp6MWD* per cent of predicted six-minute walking distance, *pre* before the six-minute walking test, *BORG* numerical measurement of dyspnoea during exercise, *SpO*_*2*_ peripheral saturation of oxygen, *ppFEV*_*1*_ percent predicted forced expiratory volume in one second, *ppFVC*_*ex*_ percent predicted forced expiratory vital capacity, *ppTLC* percent predicted total lung capacity, *PO*_*2*_ partial pressure of oxygen, *PCO*_*2*_ Partial pressure of carbon dioxide

### Symptom and functional trends across follow-up

**Fig. 2:**
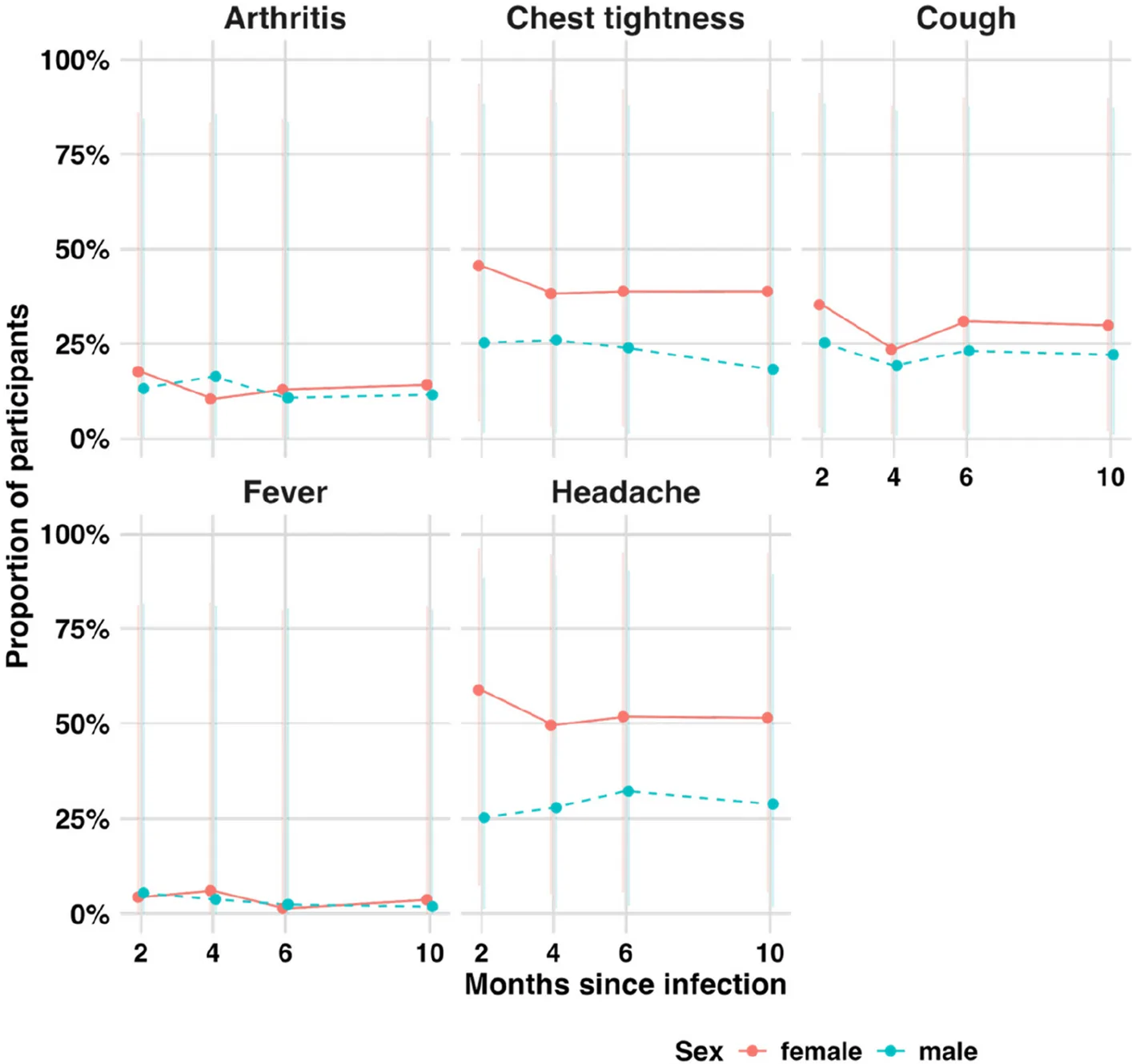
Sex-stratified prevalance of persistent symptoms across the post-infection follow-up period

**Fig. 3:**
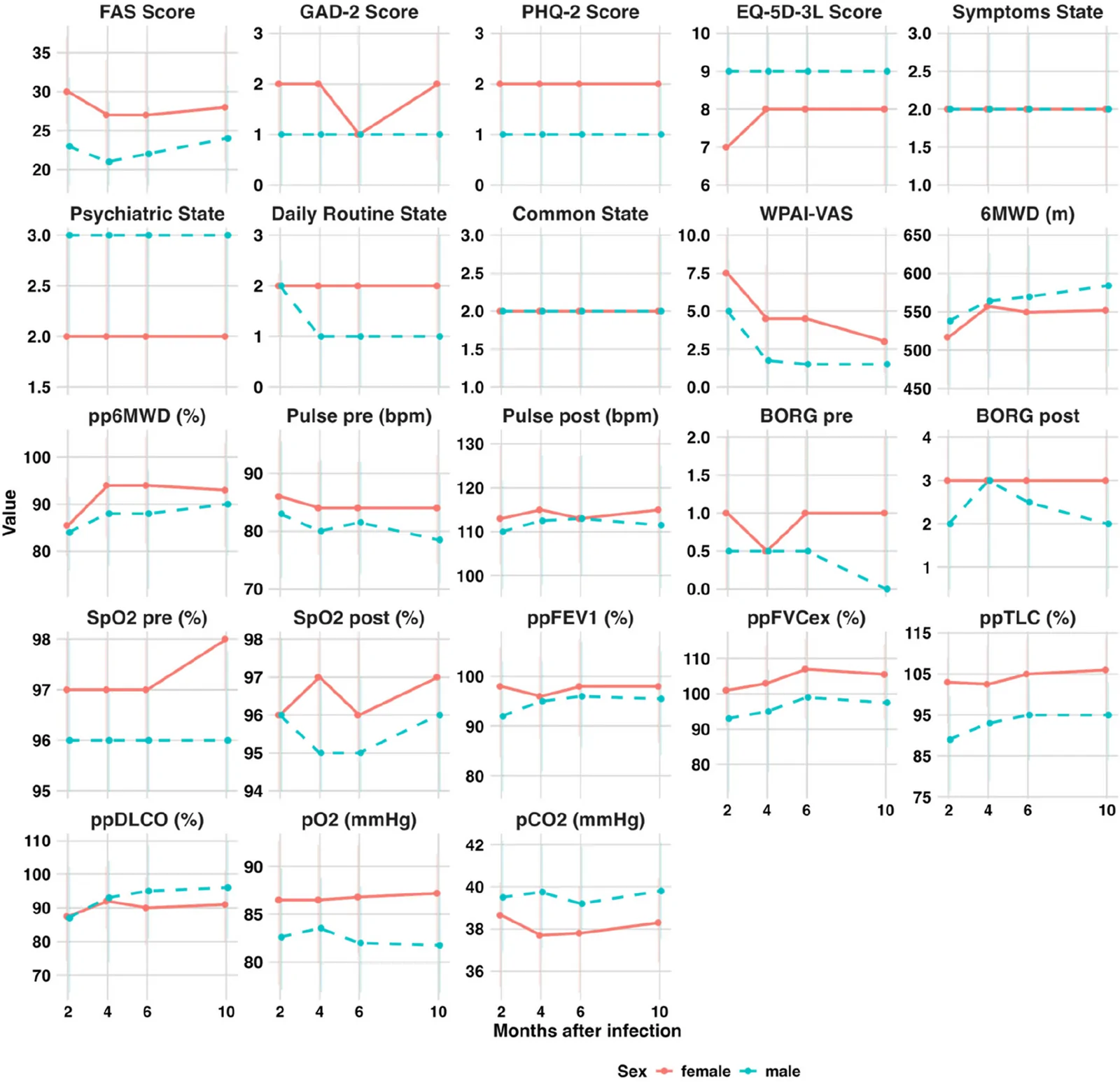
Sex-stratified trajectories of patient-reported and functional outcomes over the post-infection follow-up period. Abbreviations: FAS = Fatigue Assessment Scale, GAD2 = Generalized anxiety disorder 2- item, PHQ2 = Patient Health Questionnaire—2, EQ-5D-3L = European quality of life – 5 dimensions 3 Level Version Questionnaire, WPAI-VAS = Work Productivity and Ability Index – Visual Analog Scale, 6MWD (m) = six-minute walking distance (meters), pp6MWD = per cent of predicted six-minute walking distance, pre = before the six-minute walking test, BORG = numerical measurement of dyspnoea during exercise, SpO_2_ = peripheral saturation of oxygen, ppFEV_1_ = percent predicted forced expiratory volume in one second, ppFVC_ex_ = percent predicted forced expiratory vital capacity, ppTLC = percent predicted total lung capacity, PO_2=_ partial pressure of oxygen, PCO_2_ = partial pressure of carbon dioxide

Unadjusted trends of symptoms, PROMs, lung function and exercise capacity are provided in Figs. 2 and 3. Figure 2 presents the results for the symptoms during the different visits. Female participants complained about chest tightness at a higher proportion two, six and ten months after infection. Moreover, headache was reported more often by females at all timepoints.

PROMs in female and male participants are demonstrated in Fig. 3. In general, females reported a greater symptom burden across a range of domains than male subjects. Additional trends visible in Fig. 3 (SpO_2_, BORG dyspnoea scale, ppFCV_ex_ and pO_2_) did not reach statistical significance after adjustment and multiple-testing correction (see Table 2). All pulmonary function and blood gas results remained within clinically normal ranges.

**Fig. 4:**
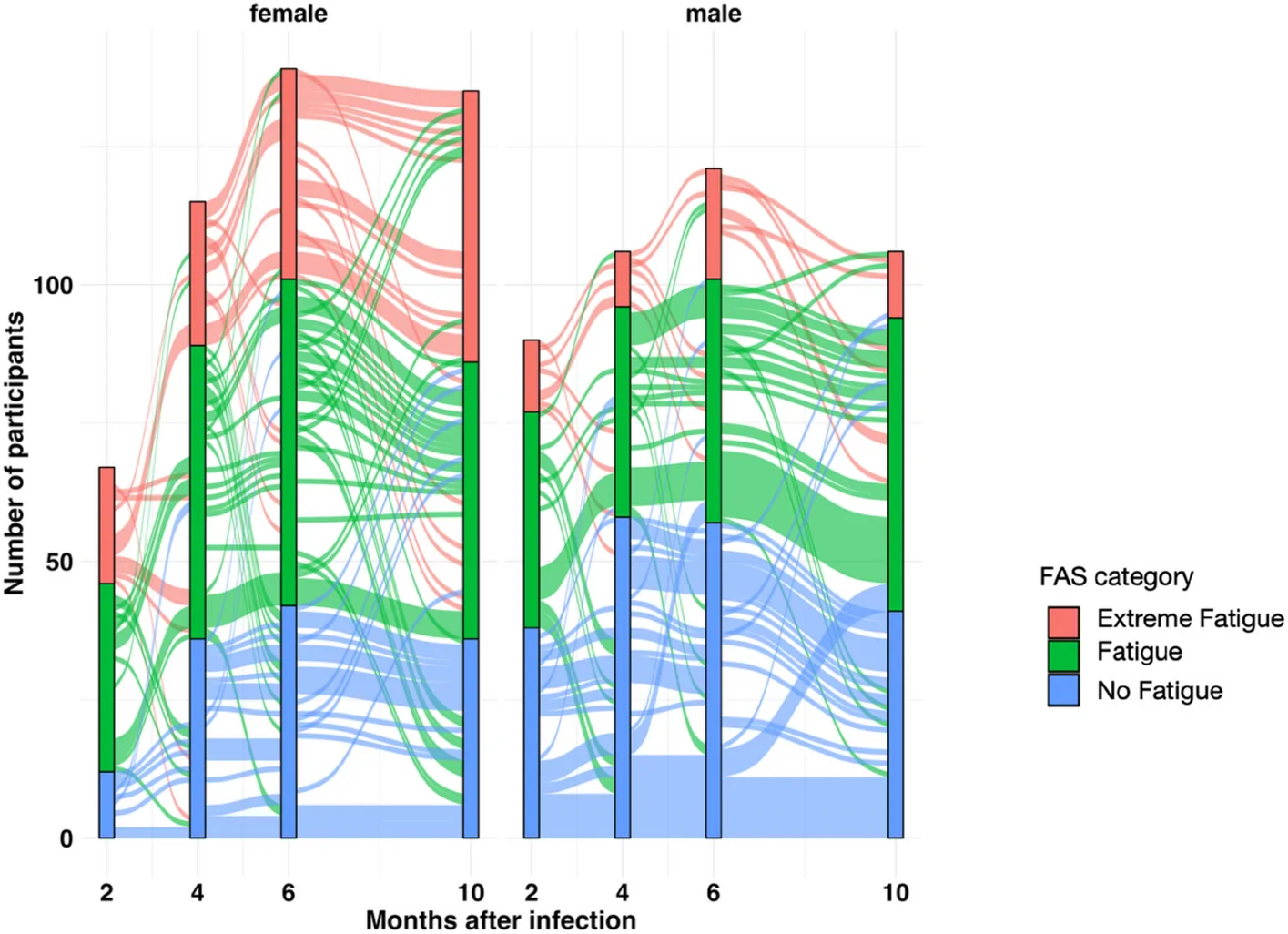
Alluvial plot of the distribution of fatigue categories based on the FAS Score (fatigue, extreme fatigue, and no fatigue), stratified by sex, across the post-infection follow-up period. Each line represents one participant. Interpretation should consider the dynamic cohort structure and differing participant availability between visits. Abbreviation: FAS = Fatigue Assessment Scale

Figure 4 illustrates the distribution of fatigue categories based on FAS scores (no fatigue, fatigue, extreme fatigue) across all follow-up appointments. Details are shown in Supplementary Table 2. It is acknowledged that there is considerable variation in the maximum number of patients across the different visits, owing to the dynamic patient cohort (see Fig. 1). Among male participants, the proportion reporting fatigue or extreme fatigue was 57.7% two months after infection and 61.3% ten months after infection (see Supplementary Table 2). Among female participants, the corresponding proportions were 82.0% two months after infection and 73.6% ten months after infection. When considering extreme fatigue separately, females demonstrated slightly higher relative frequencies ten months after infection than two months after infection (31.3% vs. 36.3%), whereas lower proportions were observed among males ten months after infection than two months after infection (14.4% vs. 11.3%).

### Logistic regression of relevant work productivity impairment

**Fig. 5:**
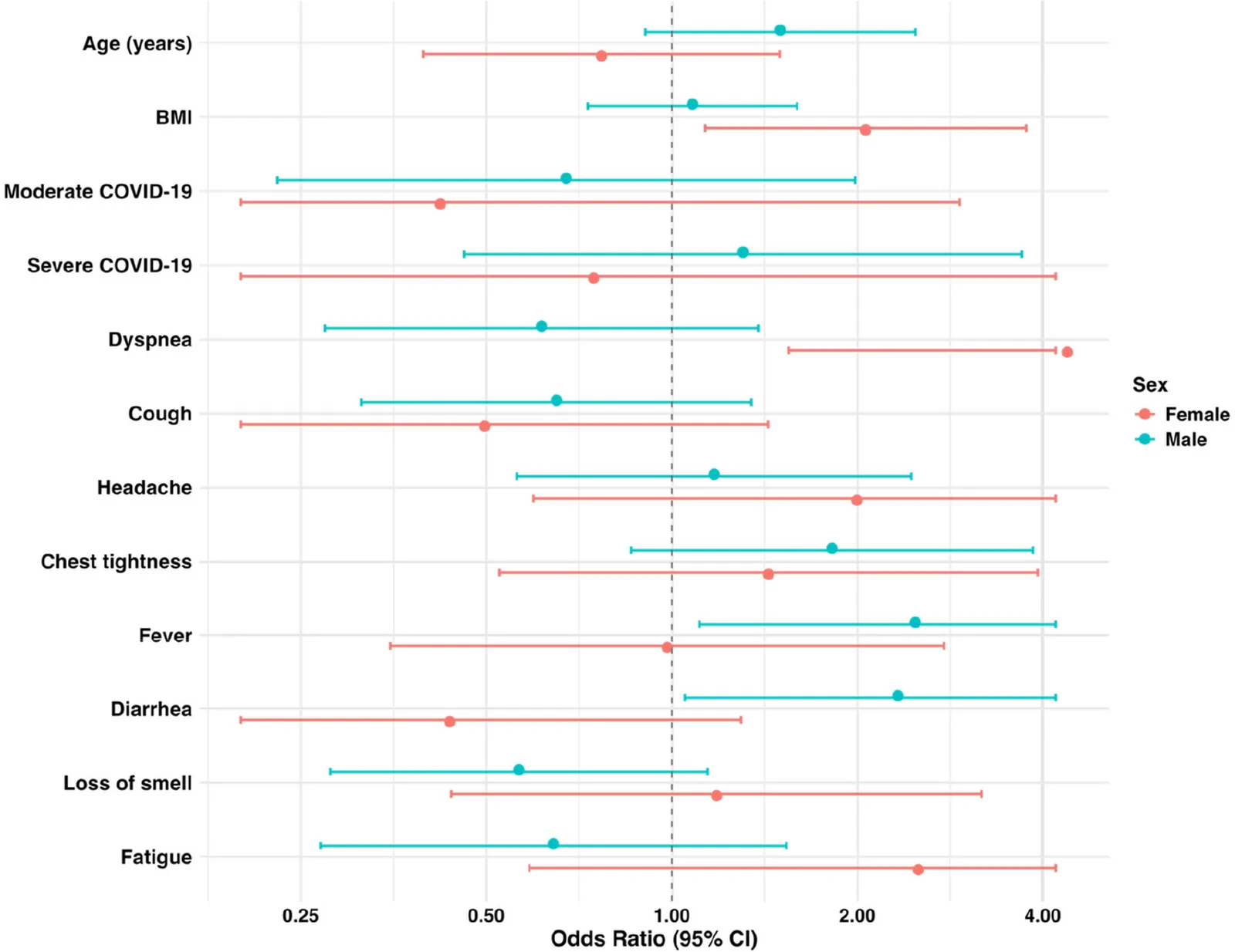
Forest plot illustrating odds ratios (OR) and 95% confidence intervals derived from binomial generalised linear mixed-effects models assessing factors associated with relevant work productivity impairment (WPAI-VAS ≥ 2). Analyses were performed separately for male and female participants. Covariates include age, body mass index (BMI), COVID-19 severity, and acute COVID-19-related symptoms. OR > 1 indicates increased odds of relevant impairment

Figure 5 illustrated the results of the regression models separately for each sex, and Supplementary Table 3 shows the statistical details. In male patients, fever (OR = 2.48, 95% CI: 1.11–5.57, *p =* 0.027) and diarrhoea (OR 2.33, 95%CI: 1.05–5.16, *p =* 0.038) during acute COVID-19 were significantly associated with higher odds of work productivity impairment (WPAI-VAS ≥ 2). In females, higher BMI (OR = 2.06, 95% CI: 1.13–3.76, *p =* 0.018) and dyspnoea during acute COVID-19 (OR = 4.38, 95% CI: 1.549–12.40, *p =* 0.005) were associated with increased odds.

To formally assess effect modification by sex, sex-by-covariate interaction terms were evaluated in a combined mixed-effects logistic regression model. After Holm adjustment, only the interaction between sex and dyspnoea remained statistically significant (adjusted *p =* 0.013), while no evidence of effect modification was observed for the remaining covariates (Supplementary Table 4).

## Discussion

This dynamic cohort study of patients with PASC found statistically robust, baseline-adjusted sex differences in a defined subset of outcomes. After accounting for age, BMI, COVID-19 severity, and comorbidities, and after Holm correction for multiple testing across all 27 pre-specified outcome comparisons, females showed higher fatigue, higher anxiety, lower quality of life, and greater work productivity impairment (both as a continuous score and as a binary indicator of clinically relevant impairment), together with differences in ppTLC, ppDLCO, and capillary pCO2. Headache and chest tightness were the only acute-COVID-19 associated PASC symptoms that remained significantly more frequent in females after adjustment. None of these differences changed significantly across the 10-month observation period (all sex-by-time interaction *p ≥* 0.41), indicating no evidence that these sex differences changed over time. In contrast, no significant sex difference was confirmed after adjustment for depressive symptoms (PHQ-2), spirometric parameters (ppFEV1, ppFVCex), sex/height-adjusted exercise capacity (pp6MWD), post-exercise dyspnoea (BORG) or SpO_2_, pO2, or most other acute-COVID-19 symptoms, despite descriptive trends visible in the raw data (Figs. 2 and 3 and Supplementary Table 2).

In the context of acute COVID-19, males were older, exhibited more severe courses and were more likely to necessitate ICU admission. They had higher BMI and more cardiovascular comorbidities, diabetes and hypertension. It is already known that males experience more severe illness in combination with the above-mentioned comorbidities [27]. However, notable variations in the reported symptoms during the acute phase were also observed, with females referring more often about headache, chest tightness, loss of smell and fatigue in our study. A Swiss study has also documented disparities in the manifestation of acute symptoms, showing that females were more likely to report anosmia/dysomia, ageusia/dysgeusia, gastrointestinal symptoms, dyspnoea, or fatigue, while males were more prone to declare fever [9]. A recent review also found differences in sexes concerning acute symptoms, pointing out females are more likely to have psychiatric disorders, ear/nose/throat (ENT) complications, musculoskeletal and respiratory symptoms. On the other hand, male patients had more renal disorders [28].

Our study and the existing literature on this topic indicate that males and females experience PASC differently. The cause of these differences is still unknown, but the subject of current research. Notably, females aged 40–54, particularly those who are premenopausal, are at a higher risk for PASC than males of the same age [1]. The increased risk may be related not only to age-related hormonal decline but also to marked hormonal fluctuations that characterise the perimenopausal transition. As described by Santoro et al., this period is associated with substantial variability in oestrogen regulation and endocrine function, which may influence immune response and susceptibility to persistent post-infectious symptoms. Clinically, symptoms of menopause and PASC are overlapping, like mood disruption, temporary cognitive dysfunction and sleep disturbances [29]. In addition, Sylvester and colleagues describe a potentially premature onset of menopause in individuals with PASC, and an associated increased risk of cardiovascular disease and osteopenia [28]. Moreover, steroid hormones, like oestrogen and progesterone, exert immunomodulatory effects [30], which may also partly explain the differing risk of developing PASC between male and female individuals. These hormonal differences may also be an explanation for the differences in psychiatric burden, as even in the general, worldwide population, the prevalence of fatigue is higher in females than in males [31, 32]. In our study, throughout the measurements over time, females reported higher rates of fatigue, and both findings, a high prevalence and females being even more at risk, are supported by several other reported studies [1, 30].

In our study, no PASC-related symptom was formally confirmed to be more frequent among males than females after adjustment for baseline imbalance and correction for multiple testing. Among the symptoms tested, only headache and chest tightness showed a statistically robust female predominance.

Work productivity impairment was significantly higher in females, both descriptively and after adjustment for baseline imbalance and correction for multiple testing (WPAI-VAS, continuous, *p =* 0.031; binary impairment threshold OR = 3.0, *p =* 0.020), and this difference did not change significantly across the ten-month follow up and did not show a significant sex-by-time interaction. In a sensitivity analysis restricted to the 46 patients with complete four-visit follow up, the odds ratio for impairment remained in the same direction (OR = 2.49) but did not reach significance (*p =* 0.135), consistent with the substantially reduced power of this small subgroup rather than a qualitatively different finding. Using generalised linear mixed-effects models incorporating sex-by-covariate interaction terms, higher BMI and dyspnoea in females, and fever and diarrhoea in males during acute COVID-19 were associated with subsequent work productivity impairment; among these, only the interaction with dyspnoea remained significant after Holm correction, indicating that this specific association differs by sex, while the other identified risk factors should be interpreted as descriptive, sex-stratified patterns.

Regarding clinical relevance, most adjusted differences were below established or estimated MCIDs. The sex differences in FAS (3.3 points) was below the established MCID of 4 points [33]. The GAD-2 has no independently validated MCID of its own, a threshold derived from the longer GAD-7 cannot be assumed to scale proportionally to this 2-item screener, so we do not report a numeric comparison for this outcome. For EQ-5D-3L, conversion to the German Time Trade-off (TTO)-based utility index [34] yielded an unadjusted mean difference of −0.030, at the lower boundary of the published MCID range (~ 0.03–0.10). Similarly, the continuous WPAI-VAS difference (1.3) was below the 20% MCID that was ourselves used, following Wu et al. [24], to define clinically relevant impairment for the binary outcome. We therefore emphasise the binary WPAI-VAS outcome (OR = 3.0), which was explicitly anchored to this threshold. Overall, statistically significant continuous-score differences should be interpreted as overall group differences between females and males rather than necessarily individually and clinically meaningful differences.

Concerning physical performance and lung function, we saw improvements in both males and females after ten months, and mean values for all lung function parameters in both sexes remained in clinically normal range throughout follow-up. Although 6MWD differed significantly between sexes throughout follow-up, the sex/height/weight-adjusted percent-predicted value (pp6MWD)—the more clinically meaningful metric, and the one used elsewhere in this manuscript—showed no significant difference after adjustment, suggesting the raw-distance difference primarily reflects anthropometric factors such as height rather than a PASC-specific functional impairment. PpTLC was significantly higher in females and ppDLCO significantly higher in males after adjustment; however, all values were within normal ranges. Spirometric parameters (ppFEV1, ppFVC_ex_) did not differ significantly. Garbsch and colleagues also reported a higher physical performance in females before starting cardiopulmonary rehabilitation, and a slightly greater increase in capability during the rehabilitation [11].

Capillary pCO_2_ was significantly lower in females throughout follow-up after adjustment, which may reflect hyperventilation, as previously reported in patients with PASC [35]. No significant sex difference was confirmed for resting pO_2_ after adjustment, although all values remained within the normal range in both sexes.

It should be noted that we only assessed the sex of the patients as documented in routine clinical records and did not evaluate gender identity. Therefore, the conclusions drawn should be interpreted exclusively in the context of sex and not gender. Sex refers to biological attributes such as chromosomal constitution, hormonal profiles and anatomy, whereas gender encompasses socially constructed roles, behaviours and identities [36]. Gebhard and colleagues have shown that gender factors are also associated with the occurrence of PASC, so gender should be taken into account in future studies.

Symptoms of the acute COVID-19 were recalled from memory at the first visit to our outpatient clinic, which leads to the possibility of recall bias. Participants may have remembered initial symptoms or exposures with varying degrees of accuracy, which could have distorted the results. In addition, pre-existing diagnoses were assessed based on available medical records from other institutions and on baseline clinical data, such as pre-COVID-19 lung function tests or blood tests, which were not available. A key further limitation is the dynamic, unbalanced cohort structure: only 46 of 433 patients (10.6%) completed all four visits, patients entered the study at different times, and each nominal visit therefore mixes newly included and patients followed up. Sex-specific attrition was formally tested by comparing the distribution of newly included, followed up, and loss to follow up status between sexes at each visit (see Supplementary Table 4): this difference was statistically significant at the 4-month visit (Holm-adjusted *p =* 0.045) but not at 2, 6, or 10 months (Holm-adjusted *p =* 0.204–0.256), indicating differential attrition by sex at one but not all follow up visits. We addressed this in three ways: first, the primary analysis used continuous days since infection, together with mixed-effects models with patient-specific random intercepts; second, sex-by-time interaction terms were tested for every outcome, none of which were significant; third, the adjusted analyses were repeated in the subgroup of patients with complete data at all four visits (*n* = 46). In this subgroup, the direction of effect was consistent with the full-cohort estimates for fatigue, anxiety, quality of life, and absolute six-minute walk distance (see Supplementary Table 6); work productivity impairment, ppTLC, ppDLCO, and pCO_2_ no longer reached significance, which is consistent with the substantially reduction of the power of this small subgroup (*n* = 46 vs. 433) rather than a absolutely different finding. Conversely, PHQ-2 (depression) reached significance in this subgroup despite not doing so in the primary analysis; given the small sample size and the number of comparisons performed, this should be interpreted as a likely chance finding rather than a confirmed sex difference. As most patients contributed data at only one or two time points, the results should not be interpreted as confirmed individual trajectories with regard to changes within a patient over the observations period. All 27 outcome comparisons between female and male participants reported in this manuscript were adjusted for the baseline imbalances identified in Table 1 (age, BMI, COVID-19 severity, and comorbidities), and sex-by-time interaction terms were formally tested and Holm-corrected across the full set of comparisons. Eleven of 27 outcomes remained significant after this adjustment and correction (see Supplementary Table 5); outcomes that did not (including depressive symptoms, spirometric parameters, pp6MWD, and most acute-COVID-19 symptoms other than headache and chest tightness) are reported as such and should not be interpreted as confirmed sex differences, despite descriptive trends visible in Figs. 2 and 3. Furthermore, as no validated WPAI-VAS cutoff exists for PASC populations, the threshold of ≥ 2 was selected pragmatically based on comparable patient cohorts [24]. Therefore, slight misclassifications cannot be excluded, and the results should be interpreted with caution accordingly. As a sensitivity analysis, the sex-stratified models for work productivity impairment were repeated at more stringent WPAI-VAS thresholds (≥ 3 and ≥ 4). At the ≥ 3 threshold, the female model converged to stable estimates and confirmed the primary finding of a significant association with acute dyspnoea (OR = 3.73, 95% CI: 1.59–8.75, *p =* 0.003) and a nominally significant association with BMI (OR = 1.11, 95% CI: 1.02–1.20, *p =* 0.015). Models for males at the ≥ 3 threshold, and for both sexes at the ≥ 4 threshold, did not converge to numerically stable estimates, reflecting an insufficient number of events per predictor at these stricter thresholds rather than a limitation of the primary findings.

SARS-CoV-2 vaccination status at first visit and educational level were assessed and reported descriptively in Table 1. However, both could not be included as additional covariates in the WPAI-VAS models due to numerical instability (quasi-complete separation) given the already high number of covariates relative to the sample size. Whether missingness in the remaining outcomes depends on symptom severity or other clinical characteristics was not formally tested beyond the sex-specific attrition analysis (see Supplementary Table 4); this remains a limitation.

Between May 2020 and May 2024, five distinct SARS-CoV-2 virus variants emerged and circulated. Early variants, such as Alpha (B.1.1.7), Beta (B.1351), and Gamma (B.1.1.28.1) were rapidly replaced by Delta (B.1.617.2), which in turn was superseded by the Omikron variant (B.1.1.529) [37]. However, the variant was not assessed individually.

Bostanghadiri et al. underscore that the evolutionary divergence among SARS-CoV-2 variants—including transmissibility, host immune responses, and clinical severity—modulates the risk and manifestation of PASC symptoms [38]. However, current evidence on variant-specific differences in the development and course of PASC remains heterogeneous and inconclusive, highlighting the need for systematic, longitudinal cohort studies stratified by infecting variant. Lastly, the monocentric approach is a further limitation.

## Conclusion

After adjustment for baseline imbalance and correction for multiple testing, this dynamic cohort study of PASC patients confirmed sex-specific differences in fatigue, anxiety, quality of life, and work productivity impairment together with differences in selected lung function parameters and capillary pCO2, none of which showed a significant change across a 10-month observation period. These findings support the need for individualised, sex-specific follow-up care focused on fatigue and psychological burden in patients with PASC. Different baseline characteristics and acute COVID-19 symptoms were associated with work productivity impairment in female and male participants, with a formally confirmed sex-by-covariate interaction for dyspnoea, suggesting distinct risk profiles that may inform individually adapted rehabilitation and supportive interventions. Further multicentre research using complete longitudinal follow-up is warranted to confirm these findings and clarify the underlying mechanisms.

## Supplementary Information

Supplementary Material 1.

Supplementary Material 2.

Supplementary Material 3.

Supplementary Material 4.

## Data Availability

The dataset supporting the conclusions of the article is available from the corresponding author upon reasonable request.

## Abbreviations

FAS: Fatigue Assessment Scale
GAD2: Generalized anxiety disorder 2- item
PHQ2: Patient Health Questionnaire - 2
EQ-5D-3L: European quality of life - 5 Dimensions 3 Level Version Questionnaire
WPAI-VAS: Work Productivity and Ability Index - Visual Analog Scale
6MWD (m): Six-minute walking distance (meters)
pp6MWD: Percent predicted six-minute walking distance
pre: Before the six-minute walking test
BORG: Numerical measurement of dyspnoea during exercise
SpO_2_: Peripheral saturation of oxygen
ppFEV_1_: Percent predicted forced expiratory volume in one second
ppFVC_ex_: Percent predicted forced expiratory vital capacity
ppTLC: Percent predicted total lung capacity
PO_2_: Partial pressure of oxygen
PCO_2_: Partial pressure of carbon dioxide

## References

[1] Shah DP, Thaweethai T, Karlson EW, et al. Sex Differences in Long COVID. JAMA Netw Open Am Med Assoc (AMA). 2025;8:e2455430. 10.1001/jamanetworkopen.2024.55430 PMC11755195 39841477

[2] Golzardi M, Hromić-Jahjefendić A, Šutković J, et al. The aftermath of COVID-19: exploring the long-term effects on organ systems. Biomedicines. 2024;12:913. 38672267 10.3390/biomedicines12040913 PMC11048001

[3] Hirschtick JL, Titus AR, Slocum E, et al. Population-based estimates of post-acute sequelae of severe acute respiratory syndrome coronavirus 2 (SARS-CoV-2) infection (PASC) prevalence and characteristics. Clin Infect Dis. 2021;73:2055–64. 34007978 10.1093/cid/ciab408 PMC8240848

[4] Soriano JB, Murthy S, Marshall JC, et al. A clinical case definition of post-COVID-19 condition by a Delphi consensus. Lancet Infect Dis. 2022;22:e102–7. 34951953 10.1016/S1473-3099(21)00703-9 PMC8691845

[5] Kautzky A, Nopp S, Gattinger D, et al. Sex differences of post-Covid patients undergoing outpatient pulmonary rehabilitation. Biol Sex Differ. 2024;15. Available from: https://bsd.biomedcentral.com/articles/10.1186/s13293-024-00609-z. Springer Science and Business Media LLC. Cited 2025 Aug 7. 10.1186/s13293-024-00609-z PMC11034076 38650012

[6] Mangion K, Morrow AJ, Sykes R, et al. Post-COVID-19 illness and associations with sex and gender. BMC Cardiovasc Disord. 2023; 23. Available from: https://bmccardiovascdisord.biomedcentral.com/articles/10.1186/s12872-023-03412-7. Springer Science and Business Media LLC. Cited 2025 Aug 7. 10.1186/s12872-023-03412-7 PMC10408208 37553628

[7] Proal AD, VanElzakker MB. Long COVID or post-acute sequelae of COVID-19 (PASC): an overview of biological factors that may contribute to persistent symptoms. Front Microbiol. 2021;12:698169. 34248921 10.3389/fmicb.2021.698169 PMC8260991

[8] Peckham H, De Gruijter NM, Raine C, et al. Male sex identified by global COVID-19 meta-analysis as a risk factor for death and ITU admission. Nat Commun. 2020;11:6317. 33298944 10.1038/s41467-020-19741-6 PMC7726563

[9] Gebhard CE, Sütsch C, Gebert P, et al. Impact of sex and gender on post-COVID-19 syndrome, Switzerland, 2020. Eurosurveillance Eur Centre Dis Control Prev (ECDC). 2024;29. Available from: https://www.eurosurveillance.org/content/10.2807/1560-7917.ES.2024.29.2.2300200. Cited 2025 July 14. 10.2807/1560-7917.ES.2024.29.2.2300200 PMC10785203 38214079

[10] Sudre CH, Murray B, Varsavsky T, et al. Attributes and predictors of long COVID. Nat Med. 2021;27:626–31. 33692530 10.1038/s41591-021-01292-y PMC7611399

[11] Garbsch R, Schäfer H, Kotewitsch M, et al. Sex-specific differences of cardiopulmonary fitness and pulmonary function in exercise-based rehabilitation of patients with long-term post-COVID-19 syndrome. BMC Med. 2024;22:446. 39379918 10.1186/s12916-024-03658-8 PMC11463035

[12] Bucciarelli V, Nasi M, Bianco F, et al. Depression pandemic and cardiovascular risk in the COVID-19 era and long COVID syndrome: Gender makes a difference. Trends Cardiovasc Med Elsevier BV. 2022;32:12–7. 10.1016/j.tcm.2021.09.009 PMC8490128 34619336

[13] Takahashi T, Ellingson MK, Wong P, et al. Sex differences in immune responses that underlie COVID-19 disease outcomes. Nature. 2020;588:315–20. 32846427 10.1038/s41586-020-2700-3 PMC7725931

[14] Kroenke K, Spitzer RL, Williams JBW. The Patient Health Questionnaire-2: Validity of a Two-Item Depression Screener. Med Care. 2003;41:1284–92. 14583691 10.1097/01.MLR.0000093487.78664.3C

[15] Kroenke K, Spitzer RL, Williams JBW, et al. Anxiety disorders in primary care: prevalence, impairment, comorbidity, and detection. Ann Intern Med. 2007;146:317–25. 17339617 10.7326/0003-4819-146-5-200703060-00004

[16] EuroQol - a new facility for the measurement of health-related quality of life. Health Policy. 1990;16:199–208. 10.1016/0168-8510(90)90421-9. 10109801

[17] Sapra A, Bhandari P, Sharma S, et al. Using Generalized Anxiety Disorder-2 (GAD-2) and GAD-7 in a Primary Care Setting. Cureus. 2020. Available from: https://www.cureus.com/articles/31476-using-generalized-anxiety-disorder-2-gad-2-and-gad-7-in-a-primary-care-setting. Cited 2025 Nov 28. 10.7759/cureus.8224 PMC7306644 32582485

[18] Arroll B, Goodyear-Smith F, Crengle S, et al. Validation of PHQ-2 and PHQ-9 to screen for major depression in the primary care population. Ann Fam Med. 2010;8:348–53. 20644190 10.1370/afm.1139 PMC2906530

[19] Xie F, Gaebel K, Perampaladas K, et al. Comparing EQ-5D valuation studies: a systematic review and methodological reporting checklist. Med Decis Making. 2014;34:8–20. 23525701 10.1177/0272989X13480852

[20] Vries J, Michielsen H, Heck GL, et al. Measuring fatigue in sarcoidosis: the Fatigue Assessment Scale (FAS). Br J Health Psychol. 2004;9:279–91. 15296678 10.1348/1359107041557048

[21] Klok FA, Boon GJAM, Barco S, et al. The Post-COVID-19 Functional Status scale: a tool to measure functional status over time after COVID-19. Eur Respir J. 2020;56:2001494. 32398306 10.1183/13993003.01494-2020 PMC7236834

[22] Pink I, Hennigs JK, Ruhl L, et al. Blood T cell phenotypes correlate with fatigue severity in post-acute sequelae of COVID-19. Infection. 2024;52:513–24 Springer Science and Business Media LLC. 37924472 10.1007/s15010-023-02114-8 PMC10954951

[23] Reilly MC, Zbrozek AS, Dukes EM. The Validity and Reproducibility of a Work Productivity and Activity Impairment Instrument. PharmacoEconomics. 1993;4:353–65 Springer Science and Business Media LLC. 10146874 10.2165/00019053-199304050-00006

[24] Wu JJ, Lin C, Sun L, et al. Minimal clinically important difference (MCID) for work productivity and activity impairment (WPAI) questionnaire in psoriasis patients. Acad Dermatol Venereol. 2019;33:318–24. 10.1111/jdv.15098 29846976

[25] Enright PL, Sherrill DL. Reference equations for the six-minute walk in healthy adults. Am J Respir Crit Care Med. 1998;158:1384–7. 9817683 10.1164/ajrccm.158.5.9710086

[26] Borg GA. Psychophysical bases of perceived exertion. Med Sci Sports Exerc. 1982;14:377–81. 7154893

[27] Bates D, Maechler M, Bolker B, et al. lme4: Linear Mixed-Effects Models using “Eigen” and S4. 2003. p. 2.0–1. Available from: https://CRAN.R-project.org/package=lme4. Cited 2026 May 19.

[28] Wickham H, Chang W, Henry L, et al. ggplot2: Create Elegant Data Visualisations Using the Grammar of Graphics. 2007. p. 4.0.3. Available from: https://CRAN.R-project.org/package=ggplot2. Cited 2026 May 19.

[29] Karagiannidis C, Mostert C, Hentschker C, et al. Case characteristics, resource use, and outcomes of 10 021 patients with COVID-19 admitted to 920 German hospitals: an observational study. Lancet Respir Med. 2020;8:853–62. 32735842 10.1016/S2213-2600(20)30316-7 PMC7386882

[30] Sylvester SV, Rusu R, Chan B, et al. Sex differences in sequelae from COVID-19 infection and in long COVID syndrome: a review. Curr Med Res Opin. 2022;38:1391–9. 35726132 10.1080/03007995.2022.2081454

[31] Santoro N, Roeca C, Peters BA, et al. The menopause transition: signs, symptoms, and management options. J Clin Endocrinol Metab. 2021;106:1–15. 33095879 10.1210/clinem/dgaa764

[32] Townsend EA, Miller VM, Prakash YS. Sex differences and sex steroids in lung health and disease. Endocr Rev. 2012;33:1–47. 22240244 10.1210/er.2010-0031 PMC3365843

[33] Chen C-W, Lee H-H, Chang S-H, et al. Risk of chronic fatigue syndrome after COVID-19: a retrospective cohort study of 3227281 patients. J Infect Public Health. 2024;17:102559. 39418958 10.1016/j.jiph.2024.102559

[34] Regitz-Zagrosek V. Sex and gender differences in health: science & society series on sex and science. EMBO Rep. 2012;13:596–603. 22699937 10.1038/embor.2012.87 PMC3388783

[35] Wood J, Tabacof L, Tosto-Mancuso J, et al. Levels of end-tidal carbon dioxide are low despite normal respiratory rate in individuals with long COVID. J Breath Res. 2022;16:017101. 10.1088/1752-7163/ac3c18 34808607

[36] Lee C, Suzuki J. COVID-19: variants, immunity, and therapeutics for non-hospitalized patients. Biomedicines. 2023;11:2055. 37509694 10.3390/biomedicines11072055 PMC10377623

[37] Bostanghadiri N, Ziaeefar P, Mofrad MG, et al. COVID-19: An Overview of SARS-CoV-2 Variants—The Current Vaccines and Drug Development. BioMed Res Int. 2023;2023:1879554 Muddassir Ali M, ed. 37674935 10.1155/2023/1879554 PMC10480030

