# Supplementary material for: Sex-specific patterns of symptom burden, psychological distress, and work productivity impairment in post-acute sequelae of COVID-19: a 10-month dynamic cohort study
Source: BMC Pulm Med. 2026 Sep 19;26:418. doi: 10.1186/s12890-026-04741-x (PMC13589870; doi:10.1186/s12890-026-04741-x)
Supplement: Supplementary file 1 [file 12890_2026_4741_MOESM1_ESM.docx]

Supplementary Table 1. Details of female and male participants newly included, follow-up and lost to follow up at each timepoint.

| Months since infection | Status | Female | Male | Holm adjusted  P value |
| --- | --- | --- | --- | --- |
| 2 |  |  |  | 0.203^1^ |
|  | Newly included | 68 | 91 |  |
| 4 |  |  |  | 0.045^1^ |
|  | Newly included | 75 | 49 |  |
|  | Followed up | 40 | 57 |  |
|  | Lost to follow up | 19 | 25 |  |
| 6 |  |  |  | 0.256^1^ |
|  | Newly included | 59 | 38 |  |
|  | Followed up | 80 | 83 |  |
|  | Lost to follow up | 34 | 23 |  |
| 10 |  |  |  | 0.256^1^ |
|  | Newly included | 35 | 18 |  |
|  | Followed up | 100 | 88 |  |
|  | Lost to follow up | 49 | 42 |  |

1. *Pearson’s Chi-squared test*
