# Supplementary material for: Sex-specific patterns of symptom burden, psychological distress, and work productivity impairment in post-acute sequelae of COVID-19: a 10-month dynamic cohort study
Source: BMC Pulm Med. 2026 Sep 19;26:418. doi: 10.1186/s12890-026-04741-x (PMC13589870; doi:10.1186/s12890-026-04741-x)
Supplement: Supplementary file 2 [file 12890_2026_4741_MOESM2_ESM.docx]

Supplementary Table 2. Differences between female and male participants at the different timepoints (two to ten months)

Two months after infection

|  | Female (N=68) | Male (N=91) | Total (N=159) |
| --- | --- | --- | --- |
| **ppFEV_1_** |  |  |  |
| Median (Q1, Q3) | 98.000 (83.750, 105.750) | 92.000 (77.000, 103.000) | 96.000 (82.000, 104.000) |
| Missings | 2 | 2 | 4 |
| **ppFEVC_ex_** |  |  |  |
| Median (Q1, Q3) | 101.000 (91.000, 109.750) | 93.000 (72.500, 103.000) | 96.000 (82.000, 107.000) |
| Missings | 2 | 0 | 2 |
| **ppTLC** |  |  |  |
| Median (Q1, Q3) | 103.000 (93.000, 109.000) | 89.000 (76.500, 99.500) | 95.000 (83.000, 106.000) |
| Missings | 2 | 0 | 2 |
| **ppDLCO** |  |  |  |
| Median (Q1, Q3) | 87.500 (74.500, 100.500) | 87.000 (65.000, 102.000) | 87.000 (71.000, 102.000) |
| Missings | 6 | 2 | 8 |
| **pp6MWD** |  |  |  |
| Median (Q1, Q3) | 85.500 (79.000, 95.500) | 84.000 (76.000, 91.500) | 85.000 (77.000, 92.500) |
| Missings | 4 | 4 | 8 |
| **6MWD (m)** |  |  |  |
| Median (Q1, Q3) | 517.000 (455.250, 573.000) | 538.000 (452.000, 596.000) | 528.000 (453.000, 586.500) |
| Missings | 4 | 4 | 8 |
| **Pulse pre 6MW** |  |  |  |
| Median (Q1, Q3) | 86.000 (76.000, 96.250) | 83.000 (72.000, 95.500) | 84.000 (75.000, 96.000) |
| Missings | 4 | 4 | 8 |
| **Pulse post 6MW** |  |  |  |
| Median (Q1, Q3) | 113.000 (102.750, 129.500) | 110.000 (97.000, 121.500) | 111.000 (99.000, 126.000) |
| Missings | 4 | 4 | 8 |
| **BORG pre 6MW** |  |  |  |
| Median (Q1, Q3) | 1.000 (0.000, 2.000) | 0.500 (0.000, 2.000) | 0.500 (0.000, 2.000) |
| Missings | 4 | 4 | 8 |
| **BORG post 6MW** |  |  |  |
| Median (Q1, Q3) | 3.000 (2.000, 4.000) | 2.000 (0.500, 3.000) | 3.000 (1.000, 4.000) |
| Missings | 4 | 4 | 8 |
| **SpO_2_ pre 6MW** |  |  |  |
| Median (Q1, Q3) | 97.000 (96.000, 98.000) | 96.000 (95.000, 98.000) | 97.000 (96.000, 98.000) |
| Missings | 4 | 4 | 8 |
| **SpO_2_ post 6MW** |  |  |  |
| Median (Q1, Q3) | 96.000 (94.000, 98.000) | 96.000 (94.000, 98.000) | 96.000 (94.000, 98.000) |
| Missings | 4 | 4 | 8 |
| **PO_2_** |  |  |  |
| Median (Q1, Q3) | 86.500 (77.600, 92.725) | 82.600 (77.150, 89.800) | 84.000 (77.150, 90.850) |
| Missings | 0 | 0 | 0 |
| **PCO_2_** |  |  |  |
| Median (Q1, Q3) | 38.650 (35.250, 40.200) | 39.500 (36.950, 42.200) | 39.000 (36.150, 41.500) |
| Missings | 0 | 0 | 0 |
| **PHQ-2 Score** |  |  |  |
| Median (Q1, Q3) | 2.000 (1.000, 3.000) | 1.000 (0.000, 2.000) | 2.000 (1.000, 2.000) |
| Missings | 0 | 0 | 0 |
| **GAD-2 Score** |  |  |  |
| Median (Q1, Q3) | 2.000 (1.000, 2.000) | 1.000 (0.000, 2.000) | 1.000 (0.000, 2.000) |
| Missings | 0 | 0 | 0 |
| **EQ-5D-3L Score** |  |  |  |
| Median (Q1, Q3) | 7.000 (6.000, 9.000) | 9.000 (7.000, 10.000) | 8.000 (6.000, 10.000) |
| Missings | 0 | 0 | 0 |
| **FAS Score** |  |  |  |
| Median (Q1, Q3) | 30.000 (26.000, 37.000) | 23.000 (18.000, 31.750) | 27.000 (19.000, 34.000) |
| Missings | 1 | 1 | 2 |
| **WPAI-VAS** |  |  |  |
| Median (Q1, Q3) | 7.500 (4.500, 10.000) | 5.000 (2.125, 8.375) | 6.500 (2.500, 10.000) |
| Missings | 25 | 33 | 58 |
| **Symptom state** |  |  |  |
| Median (Q1, Q3) | 2.000 (1.000, 3.000) | 2.000 (2.000, 3.000) | 2.000 (1.000, 3.000) |
| Missings | 0 | 0 | 0 |
| **Psychiatric state** |  |  |  |
| Median (Q1, Q3) | 2.000 (2.000, 3.000) | 3.000 (2.000, 3.000) | 3.000 (2.000, 3.000) |
| Missings | 0 | 0 | 0 |
| **Daily routine state** |  |  |  |
| Median (Q1, Q3) | 2.000 (1.000, 2.250) | 2.000 (1.000, 2.500) | 2.000 (1.000, 2.500) |
| Missings | 0 | 0 | 0 |
| **Common state** |  |  |  |
| Median (Q1, Q3) | 2.000 (1.000, 2.000) | 2.000 (1.000, 2.000) | 2.000 (1.000, 2.000) |
| Missings | 0 | 0 | 0 |
| **Headache** |  |  |  |
| Yes | 40 (58.8%) | 23 (25.3%) | 63 (39.6%) |
| No | 28 (41.2%) | 68 (74.7%) | 96 (60.4%) |
| Missings | 0 | 0 | 0 |
| **Chest tightness** |  |  |  |
| Yes | 31 (45.6%) | 23 (25.3%) | 54 (34.0%) |
| No | 37 (54.4%) | 68 (74.7%) | 105 (66.0%) |
| Missings | 0 | 0 | 0 |
| **Arthritis** |  |  |  |
| Yes | 12 (17.6%) | 12 (13.2%) | 24 (15.1%) |
| No | 56 (82.4%) | 79 (86.8%) | 135 (84.9%) |
| Missings | 0 | 0 | 0 |
| **Cough** |  |  |  |
| Yes | 24 (35.3%) | 23 (25.3%) | 47 (29.6%) |
| No | 44 (64.7%) | 68 (74.7%) | 112 (70.4%) |
| Missings | 0 | 0 | 0 |
| **Fever** |  |  |  |
| Yes | 3 (4.4%) | 5 (5.5%) | 8 (5.0%) |
| No | 65 (95.6%) | 86 (94.5%) | 151 (95.0%) |
| Missings | 0 | 0 | 0 |
| **FAS category** |  |  |  |
| extreme Fatigue | 21 (31.3%) | 13 (14.4%) | 34 (21.7%) |
| Fatigue | 34 (50.7%) | 39 (43.3%) | 73 (46.5%) |
| No Fatigue | 12 (17.9%) | 38 (42.2%) | 50 (31.8%) |
| Missings | 1 | 1 | 2 |

1. Wilcoxon rank sum test
2. Pearson’s Chi-squared test

Abbreviations: FAS= Fatigue Assessment Scale, GAD2= Generalized anxiety disorder 2- item, PHQ2 = Patient Health Questionnaire - 2, EQ-5D-3L= European quality of life – 5 dimensions 3 Level Version Questionnaire, WPAI-VAS= Work Productivity and Ability Index – Visual Analog Scale, 6MWD (m)= six-minute walking distance (meters), pp6MWD= per cent of predicted six-minute walking distance, pre= before the six-minute walking test, BORG= numerical measurement of dyspnoea during exercise, SpO_2_= peripheral saturation of oxygen, ppFEV_1_= percent predicted forced expiratory volume in one second, ppFVC_ex_= percent predicted forced expiratory vital capacity, ppTLC= percent predicted total lung capacity, PO_2=_ partial pressure of oxygen, PCO_2_= partial pressure of carbon dioxide.

Four months after infection

|  | Female (N=115) | Male (N=106) | Total (N=221) |
| --- | --- | --- | --- |
| **ppFEV_1_** |  |  |  |
| Median (Q1, Q3) | 96.000 (87.500, 104.000) | 95.000 (81.000, 103.000) | 95.000 (86.000, 104.000) |
| Missings | 0 | 2 | 2 |
| **ppFEVC_ex_** |  |  |  |
| Median (Q1, Q3) | 103.000 (93.500, 113.500) | 95.000 (78.000, 106.750) | 100.000 (87.000, 111.000) |
| Missings | 0 | 0 | 0 |
| **ppTLC** |  |  |  |
| Median (Q1, Q3) | 102.500 (97.000, 111.750) | 93.000 (79.000, 102.000) | 99.000 (87.500, 108.000) |
| Missings | 1 | 1 | 2 |
| **ppDLCO** |  |  |  |
| Median (Q1, Q3) | 92.000 (84.000, 102.000) | 93.000 (74.000, 104.000) | 93.000 (80.750, 104.000) |
| Missings | 2 | 3 | 5 |
| **Pp6MWD** |  |  |  |
| Median (Q1, Q3) | 94.000 (83.000, 104.000) | 88.000 (77.000, 96.000) | 90.000 (79.250, 100.750) |
| Missings | 2 | 1 | 3 |
| **6MWD (m)** |  |  |  |
| Median (Q1, Q3) | 557.000 (495.000, 606.000) | 564.000 (464.000, 627.000) | 561.000 (489.250, 612.000) |
| Missings | 2 | 1 | 3 |
| **Pulse pre 6MW** |  |  |  |
| Median (Q1, Q3) | 84.000 (76.000, 92.000) | 80.000 (70.000, 92.250) | 82.000 (74.000, 92.000) |
| Missings | 2 | 2 | 4 |
| **Pulse post 6MW** |  |  |  |
| Median (Q1, Q3) | 115.000 (104.000, 129.000) | 112.500 (98.000, 127.250) | 114.000 (101.000, 128.000) |
| Missings | 2 | 2 | 4 |
| **BORG pre 6MW** |  |  |  |
| Median (Q1, Q3) | 0.500 (0.000, 2.000) | 0.500 (0.000, 2.000) | 0.500 (0.000, 2.000) |
| Missings | 2 | 2 | 4 |
| **BORG post 6MW** |  |  |  |
| Median (Q1, Q3) | 3.000 (2.000, 4.000) | 3.000 (1.000, 4.000) | 3.000 (1.000, 4.000) |
| Missings | 2 | 2 | 4 |
| **SpO_2_ pre 6MW** |  |  |  |
| Median (Q1, Q3) | 97.000 (96.000, 98.000) | 96.000 (95.000, 97.000) | 97.000 (95.000, 98.000) |
| Missings | 2 | 2 | 4 |
| **SpO_2_ post 6MW** |  |  |  |
| Median (Q1, Q3) | 97.000 (95.000, 98.000) | 95.000 (94.000, 97.000) | 96.000 (95.000, 97.000) |
| Missings | 2 | 2 | 4 |
| **PO_2_** |  |  |  |
| Median (Q1, Q3) | 86.500 (79.925, 92.225) | 83.550 (76.900, 88.775) | 85.100 (78.375, 90.600) |
| Missings | 1 | 0 | 1 |
| **PCO_2_** |  |  |  |
| Median (Q1, Q3) | 37.700 (35.350, 40.000) | 39.750 (37.100, 42.000) | 38.000 (36.000, 41.000) |
| Missings | 0 | 0 | 0 |
| **PHQ-2 Score** |  |  |  |
| Median (Q1, Q3) | 2.000 (1.000, 2.000) | 1.000 (0.000, 2.000) | 1.000 (0.000, 2.000) |
| Missings | 0 | 2 | 2 |
| **GAD-2 Score** |  |  |  |
| Median (Q1, Q3) | 2.000 (1.000, 2.000) | 1.000 (0.000, 2.000) | 1.000 (0.000, 2.000) |
| Missings | 0 | 2 | 2 |
| **EQ-5D-3L Score** |  |  |  |
| Median (Q1, Q3) | 8.000 (7.000, 9.000) | 9.000 (7.000, 10.000) | 8.000 (7.000, 10.000) |
| Missings | 0 | 2 | 2 |
| **FAS Score** |  |  |  |
| Median (Q1, Q3) | 27.000 (20.500, 34.000) | 21.000 (18.000, 28.750) | 24.000 (18.000, 32.000) |
| Missings | 0 | 0 | 0 |
| **WPAI-VAS** |  |  |  |
| Median (Q1, Q3) | 4.500 (1.250, 8.000) | 1.750 (0.000, 5.000) | 3.000 (0.500, 7.500) |
| Missings | 32 | 40 | 72 |
| **Symptom state** |  |  |  |
| Median (Q1, Q3) | 2.000 (1.000, 3.000) | 2.000 (2.000, 3.000) | 2.000 (1.000, 3.000) |
| Missings | 0 | 1 | 1 |
| **Psychiatric state** |  |  |  |
| Median (Q1, Q3) | 2.000 (2.000, 3.000) | 3.000 (2.000, 3.000) | 3.000 (2.000, 3.000) |
| Missings | 0 | 1 | 1 |
| **Daily routine state** |  |  |  |
| Median (Q1, Q3) | 2.000 (1.000, 2.000) | 1.000 (0.000, 2.000) | 1.000 (1.000, 2.000) |
| Missings | 0 | 1 | 1 |
| **Common state** |  |  |  |
| Median (Q1, Q3) | 2.000 (1.000, 2.000) | 2.000 (1.000, 3.000) | 2.000 (1.000, 3.000) |
| Missings | 0 | 1 | 1 |
| **Headache** |  |  |  |
| Yes | 58 (50.4%) | 30 (28.8%) | 88 (40.2%) |
| No | 57 (49.6%) | 74 (71.2%) | 131 (59.8%) |
| Missings | 0 | 2 | 2 |
| **Chest tightness** |  |  |  |
| Yes | 46 (40.0%) | 28 (26.9%) | 74 (33.8%) |
| No | 69 (60.0%) | 76 (73.1%) | 145 (66.2%) |
| Missings | 0 | 2 | 2 |
| **Arthritis** |  |  |  |
| Yes | 12 (10.4%) | 17 (16.3%) | 29 (13.2%) |
| No | 103 (89.6%) | 87 (83.7%) | 190 (86.8%) |
| Missings | 0 | 2 | 2 |
| **Cough** |  |  |  |
| Yes | 30 (26.1%) | 22 (21.2%) | 52 (23.7%) |
| No | 85 (73.9%) | 82 (78.8%) | 167 (76.3%) |
| Missings | 0 | 2 | 2 |
| **Fever** |  |  |  |
| Yes | 7 (6.1%) | 5 (4.8%) | 12 (5.5%) |
| No | 108 (93.9%) | 99 (95.2%) | 207 (94.5%) |
| Missings | 0 | 2 | 2 |
| **FAS category** |  |  |  |
| extreme Fatigue | 26 (22.6%) | 10 (9.4%) | 36 (16.3%) |
| Fatigue | 53 (46.1%) | 38 (35.8%) | 91 (41.2%) |
| No Fatigue | 36 (31.3%) | 58 (54.7%) | 94 (42.5%) |
| Missings | 0 | 0 | 0 |

1. Wilcoxon rank sum test
2. Pearson’s Chi-squared test

Abbreviations: FAS= Fatigue Assessment Scale, GAD2= Generalized anxiety disorder 2- item, PHQ2 = Patient Health Questionnaire - 2, EQ-5D-3L= European quality of life – 5 dimensions 3 Level Version Questionnaire, WPAI-VAS= Work Productivity and Ability Index – Visual Analog Scale, 6MWD (m)= six-minute walking distance (meters), pp6MWD= per cent of predicted six-minute walking distance, pre= before the six-minute walking test, BORG= numerical measurement of dyspnoea during exercise, SpO_2_= peripheral saturation of oxygen, ppFEV_1_= percent predicted forced expiratory volume in one second, ppFVC_ex_= percent predicted forced expiratory vital capacity, ppTLC= percent predicted total lung capacity, PO_2=_ partial pressure of oxygen, PCO_2_= partial pressure of carbon dioxide.

Six months after infection

|  | Female (N=139) | Male (N=121) | Total (N=260) |
| --- | --- | --- | --- |
| **ppFEV_1_** |  |  |  |
| Median (Q1, Q3) | 98.000 (88.000, 107.500) | 96.000 (85.750, 107.000) | 97.000 (87.000, 107.000) |
| Missings | 0 | 1 | 1 |
| **ppFEVC_ex_** |  |  |  |
| Median (Q1, Q3) | 107.000 (93.000, 115.500) | 99.000 (86.000, 108.000) | 102.000 (90.000, 114.000) |
| Missings | 0 | 1 | 1 |
| **ppTLC** |  |  |  |
| Median (Q1, Q3) | 105.000 (96.000, 113.500) | 95.000 (84.000, 103.500) | 101.000 (90.000, 109.000) |
| Missings | 0 | 2 | 2 |
| **ppDLCO** |  |  |  |
| Median (Q1, Q3) | 90.000 (79.000, 99.000) | 95.000 (81.000, 108.500) | 92.000 (79.000, 103.000) |
| Missings | 2 | 2 | 4 |
| **Pp6MWD** |  |  |  |
| Median (Q1, Q3) | 94.000 (81.750, 104.250) | 88.000 (72.000, 97.250) | 91.000 (79.750, 102.000) |
| Missings | 3 | 5 | 8 |
| **6MWD (m)** |  |  |  |
| Median (Q1, Q3) | 549.500 (482.000, 607.000) | 569.500 (453.000, 636.000) | 555.000 (462.750, 622.250) |
| Missings | 3 | 5 | 8 |
| **Pulse pre 6MW** |  |  |  |
| Median (Q1, Q3) | 84.000 (76.000, 92.000) | 81.500 (72.750, 92.000) | 83.000 (74.000, 92.000) |
| Missings | 3 | 5 | 8 |
| **Pulse post 6MW** |  |  |  |
| Median (Q1, Q3) | 113.000 (103.000, 127.000) | 113.000 (100.000, 127.250) | 113.000 (102.000, 127.000) |
| Missings | 3 | 5 | 8 |
| **BORG pre 6MW** |  |  |  |
| Median (Q1, Q3) | 1.000 (0.000, 2.000) | 0.500 (0.000, 2.000) | 0.500 (0.000, 2.000) |
| Missings | 4 | 5 | 9 |
| **BORG post 6MW** |  |  |  |
| Median (Q1, Q3) | 3.000 (2.000, 4.000) | 2.500 (1.000, 3.250) | 3.000 (1.000, 4.000) |
| Missings | 4 | 5 | 9 |
| **SpO_2_ pre 6MW** |  |  |  |
| Median (Q1, Q3) | 97.000 (96.000, 98.000) | 96.000 (95.000, 97.000) | 97.000 (95.000, 98.000) |
| Missings | 3 | 5 | 8 |
| **SpO_2_ post 6MW** |  |  |  |
| Median (Q1, Q3) | 96.000 (95.000, 98.000) | 95.000 (94.000, 97.000) | 96.000 (94.000, 98.000) |
| Missings | 3 | 5 | 8 |
| **PO_2_** |  |  |  |
| Median (Q1, Q3) | 86.800 (80.950, 92.200) | 82.000 (76.875, 87.875) | 84.300 (78.300, 90.700) |
| Missings | 0 | 1 | 1 |
| **PCO_2_** |  |  |  |
| Median (Q1, Q3) | 37.800 (35.000, 40.000) | 39.200 (38.000, 41.925) | 38.300 (36.450, 40.400) |
| Missings | 0 | 1 | 1 |
| **PHQ-2 Score** |  |  |  |
| Median (Q1, Q3) | 2.000 (1.000, 2.000) | 1.000 (0.000, 2.000) | 2.000 (0.000, 2.000) |
| Missings | 0 | 0 | 0 |
| **GAD-2 Score** |  |  |  |
| Median (Q1, Q3) | 1.000 (0.000, 2.000) | 1.000 (0.000, 2.000) | 1.000 (0.000, 2.000) |
| Missings | 0 | 0 | 0 |
| **EQ-5D-3L Score** |  |  |  |
| Median (Q1, Q3) | 8.000 (7.000, 9.000) | 9.000 (7.000, 10.000) | 8.000 (7.000, 10.000) |
| Missings | 0 | 0 | 0 |
| **FAS Score** |  |  |  |
| Median (Q1, Q3) | 27.000 (19.000, 35.000) | 22.000 (18.000, 28.000) | 25.000 (19.000, 33.000) |
| Missings | 0 | 0 | 0 |
| **WPAI-VAS** |  |  |  |
| Median (Q1, Q3) | 4.500 (1.000, 7.500) | 1.500 (0.000, 5.000) | 3.000 (0.500, 7.000) |
| Missings | 39 | 48 | 87 |
| **Symptom state** |  |  |  |
| Median (Q1, Q3) | 2.000 (1.000, 3.000) | 2.000 (2.000, 3.000) | 2.000 (1.000, 3.000) |
| Missings | 0 | 4 | 4 |
| **Psychiatric state** |  |  |  |
| Median (Q1, Q3) | 2.000 (1.500, 3.000) | 3.000 (2.000, 3.000) | 2.000 (2.000, 3.000) |
| Missings | 0 | 4 | 4 |
| **Daily routine state** |  |  |  |
| Median (Q1, Q3) | 2.000 (1.000, 2.000) | 1.000 (0.000, 2.000) | 1.000 (1.000, 2.000) |
| Missings | 0 | 4 | 4 |
| **Common state** |  |  |  |
| Median (Q1, Q3) | 2.000 (1.000, 2.500) | 2.000 (1.000, 3.000) | 2.000 (1.000, 3.000) |
| Missings | 0 | 4 | 4 |
| **Headache** |  |  |  |
| Yes | 73 (52.5%) | 39 (32.2%) | 112 (43.1%) |
| No | 66 (47.5%) | 82 (67.8%) | 148 (56.9%) |
| Missings | 0 | 0 | 0 |
| **Chest tightness** |  |  |  |
| Yes | 55 (39.6%) | 29 (24.0%) | 84 (32.3%) |
| No | 84 (60.4%) | 92 (76.0%) | 176 (67.7%) |
| Missings | 0 | 0 | 0 |
| **Arthritis** |  |  |  |
| Yes | 18 (12.9%) | 13 (10.7%) | 31 (11.9%) |
| No | 121 (87.1%) | 108 (89.3%) | 229 (88.1%) |
| Missings | 0 | 0 | 0 |
| **Cough** |  |  |  |
| Yes | 45 (32.4%) | 28 (23.1%) | 73 (28.1%) |
| No | 94 (67.6%) | 93 (76.9%) | 187 (71.9%) |
| Missings | 0 | 0 | 0 |
| **Fever** |  |  |  |
| Yes | 2 (1.4%) | 3 (2.5%) | 5 (1.9%) |
| No | 137 (98.6%) | 118 (97.5%) | 255 (98.1%) |
| Missings | 0 | 0 | 0 |
| **FAS category** |  |  |  |
| extreme Fatigue | 38 (27.3%) | 20 (16.5%) | 58 (22.3%) |
| Fatigue | 59 (42.4%) | 44 (36.4%) | 103 (39.6%) |
| No Fatigue | 42 (30.2%) | 57 (47.1%) | 99 (38.1%) |
| Missings | 0 | 0 | 0 |

1. Wilcoxon rank sum test
2. Pearson’s Chi-squared test

Abbreviations: FAS= Fatigue Assessment Scale, GAD2= Generalized anxiety disorder 2- item, PHQ2 = Patient Health Questionnaire - 2, EQ-5D-3L= European quality of life – 5 dimensions 3 Level Version Questionnaire, WPAI-VAS= Work Productivity and Ability Index – Visual Analog Scale, 6MWD (m)= six-minute walking distance (meters), pp6MWD= per cent of predicted six-minute walking distance, pre= before the six-minute walking test, BORG= numerical measurement of dyspnoea during exercise, SpO_2_= peripheral saturation of oxygen, ppFEV_1_= percent predicted forced expiratory volume in one second, ppFVC_ex_= percent predicted forced expiratory vital capacity, ppTLC= percent predicted total lung capacity, PO_2=_ partial pressure of oxygen, PCO_2_= partial pressure of carbon dioxide.

Ten months after infection

|  | female (N=135) | male (N=106) | Total (N=241) |
| --- | --- | --- | --- |
| **ppFEV_1_** |  |  |  |
| Median (Q1, Q3) | 98.000 (86.750, 106.000) | 95.500 (84.250, 105.000) | 97.000 (85.250, 106.000) |
| Missings | 3 | 4 | 7 |
| **ppFEVC_ex_** |  |  |  |
| Median (Q1, Q3) | 105.500 (93.000, 114.000) | 97.500 (85.000, 107.000) | 102.000 (88.000, 111.000) |
| Missings | 3 | 4 | 7 |
| **ppTLC** |  |  |  |
| Median (Q1, Q3) | 106.000 (95.000, 113.500) | 95.000 (84.000, 103.000) | 101.000 (90.000, 110.000) |
| Missings | 4 | 5 | 9 |
| **ppDLCO** |  |  |  |
| Median (Q1, Q3) | 91.000 (80.000, 101.000) | 96.000 (81.000, 110.000) | 93.000 (80.250, 105.750) |
| Missings | 6 | 5 | 11 |
| **Pp6MWD** |  |  |  |
| Median (Q1, Q3) | 93.000 (81.000, 103.000) | 90.000 (79.250, 99.000) | 91.500 (80.250, 102.000) |
| Missings | 7 | 8 | 15 |
| **6MWD (m)** |  |  |  |
| Median (Q1, Q3) | 552.000 (471.750, 595.250) | 584.000 (479.000, 649.750) | 561.000 (472.750, 620.750) |
| Missings | 7 | 8 | 15 |
| **Pulse pre 6MW** |  |  |  |
| Median (Q1, Q3) | 84.000 (74.500, 93.000) | 78.500 (71.000, 89.750) | 81.000 (71.000, 92.000) |
| Missings | 8 | 8 | 16 |
| **Pulse post 6MW** |  |  |  |
| Median (Q1, Q3) | 115.000 (100.500, 131.500) | 111.500 (97.250, 125.000) | 114.000 (99.000, 129.000) |
| Missings | 8 | 8 | 16 |
| **BORG pre 6MW** |  |  |  |
| Median (Q1, Q3) | 1.000 (0.000, 2.000) | 0.000 (0.000, 2.000) | 0.500 (0.000, 2.000) |
| Missings | 8 | 9 | 17 |
| **BORG post 6MW** |  |  |  |
| Median (Q1, Q3) | 3.000 (2.000, 4.000) | 2.000 (0.500, 3.000) | 3.000 (1.000, 3.000) |
| Missings | 9 | 9 | 18 |
| **SpO_2_ pre 6MW** |  |  |  |
| Median (Q1, Q3) | 98.000 (97.000, 98.000) | 96.000 (95.000, 97.750) | 97.000 (96.000, 98.000) |
| Missings | 8 | 8 | 16 |
| **SpO_2_ post 6MW** |  |  |  |
| Median (Q1, Q3) | 97.000 (95.000, 98.000) | 96.000 (94.000, 97.000) | 97.000 (95.000, 98.000) |
| Missings | 8 | 8 | 16 |
| **PO_2_** |  |  |  |
| Median (Q1, Q3) | 87.200 (81.750, 91.250) | 81.750 (76.850, 87.000) | 85.000 (79.100, 89.500) |
| Missings | 4 | 4 | 8 |
| **PCO_2_** |  |  |  |
| Median (Q1, Q3) | 38.300 (36.450, 40.400) | 39.800 (37.550, 42.150) | 39.000 (36.700, 41.100) |
| Missings | 4 | 4 | 8 |
| **PHQ-2 Score** |  |  |  |
| Median (Q1, Q3) | 2.000 (1.000, 3.000) | 1.000 (0.000, 2.000) | 2.000 (0.000, 3.000) |
| Missings | 0 | 2 | 2 |
| **GAD-2 Score** |  |  |  |
| Median (Q1, Q3) | 2.000 (0.500, 3.000) | 1.000 (0.000, 2.000) | 1.000 (0.000, 2.000) |
| Missings | 0 | 2 | 2 |
| **EQ-5D-3L Score** |  |  |  |
| Median (Q1, Q3) | 8.000 (6.000, 9.000) | 9.000 (6.000, 10.000) | 8.000 (6.000, 10.000) |
| Missings | 0 | 2 | 2 |
| **FAS Score** |  |  |  |
| Median (Q1, Q3) | 28.000 (21.000, 37.500) | 24.000 (18.250, 30.750) | 26.000 (20.000, 35.000) |
| Missings | 0 | 0 | 0 |
| **WPAI-VAS** |  |  |  |
| Median (Q1, Q3) | 3.000 (1.000, 7.500) | 1.500 (0.000, 5.000) | 2.500 (0.500, 7.000) |
| Missings | 38 | 34 | 72 |
| **Symptom state** |  |  |  |
| Median (Q1, Q3) | 2.000 (1.000, 3.000) | 2.000 (2.000, 3.000) | 2.000 (1.000, 3.000) |
| Missings | 5 | 6 | 11 |
| **Psychiatric state** |  |  |  |
| Median (Q1, Q3) | 2.000 (2.000, 3.000) | 3.000 (2.000, 3.000) | 2.000 (2.000, 3.000) |
| Missings | 5 | 6 | 11 |
| **Daily routine state** |  |  |  |
| Median (Q1, Q3) | 2.000 (1.000, 3.000) | 1.000 (0.000, 3.000) | 2.000 (1.000, 3.000) |
| Missings | 5 | 6 | 11 |
| **Common state** |  |  |  |
| Median (Q1, Q3) | 2.000 (1.000, 3.000) | 2.000 (1.000, 3.000) | 2.000 (1.000, 3.000) |
| Missings | 5 | 6 | 11 |
| **Headache** |  |  |  |
| Yes | 76 (56.7%) | 32 (30.8%) | 108 (45.4%) |
| No | 58 (43.3%) | 72 (69.2%) | 130 (54.6%) |
| Missings | 1 | 2 | 3 |
| **Chest tightness** |  |  |  |
| Yes | 57 (42.5%) | 20 (19.2%) | 77 (32.4%) |
| No | 77 (57.5%) | 84 (80.8%) | 161 (67.6%) |
| Missings | 1 | 2 | 3 |
| **Arthritis** |  |  |  |
| Yes | 20 (14.9%) | 13 (12.5%) | 33 (13.9%) |
| No | 114 (85.1%) | 91 (87.5%) | 205 (86.1%) |
| Missings | 1 | 2 | 3 |
| **Cough** |  |  |  |
| Yes | 44 (32.8%) | 23 (22.1%) | 67 (28.2%) |
| No | 90 (67.2%) | 81 (77.9%) | 171 (71.8%) |
| Missings | 1 | 2 | 3 |
| **Fever** |  |  |  |
| Yes | 6 (4.5%) | 2 (1.9%) | 8 (3.4%) |
| No | 128 (95.5%) | 102 (98.1%) | 230 (96.6%) |
| Missings | 1 | 2 | 3 |
| **FAS category** |  |  |  |
| extreme Fatigue | 49 (36.3%) | 12 (11.3%) | 61 (25.3%) |
| Fatigue | 50 (37.0%) | 53 (50.0%) | 103 (42.7%) |
| No Fatigue | 36 (26.7%) | 41 (38.7%) | 77 (32.0%) |
| Missings | 0 | 0 | 0 |

1. Wilcoxon rank sum test
2. Pearson’s Chi-squared test

Abbreviations: FAS= Fatigue Assessment Scale, GAD2= Generalized anxiety disorder 2- item, PHQ2 = Patient Health Questionnaire - 2, EQ-5D-3L= European quality of life – 5 dimensions 3 Level Version Questionnaire, WPAI-VAS= Work Productivity and Ability Index – Visual Analog Scale, 6MWD (m)= six-minute walking distance (meters), pp6MWD= per cent of predicted six-minute walking distance, pre= before the six-minute walking test, BORG= numerical measurement of dyspnoea during exercise, SpO_2_= peripheral saturation of oxygen, ppFEV_1_= percent predicted forced expiratory volume in one second, ppFVC_ex_= percent predicted forced expiratory vital capacity, ppTLC= percent predicted total lung capacity, PO_2=_ partial pressure of oxygen, PCO_2_= partial pressure of carbon dioxide.
