# Supplementary material for: Sex-specific patterns of symptom burden, psychological distress, and work productivity impairment in post-acute sequelae of COVID-19: a 10-month dynamic cohort study
Source: BMC Pulm Med. 2026 Sep 19;26:418. doi: 10.1186/s12890-026-04741-x (PMC13589870; doi:10.1186/s12890-026-04741-x)
Supplement: Supplementary file 3 [file 12890_2026_4741_MOESM3_ESM.docx]

*Supplementary Table 3. Details of the logistic regression of WPAI among male and female participants*

# 1. *Logistic Regression analysis of WPAI among female participants*

| **Variable** | **Odds Ratio** | **95% CI** | **p-value** |
| --- | --- | --- | --- |
| Intercept | 0.800 | 0.127–5.020 | 0.810 |
| Age (years) | 0.770 | 0.396–1.497 | 0.440 |
| BMI | 2.063 | 1.133–3.759 | 0.018 |
| Severe COVID-19 | 0.421 | 0.061–2.931 | 0.382 |
| COVID-19 ICU | 0.747 | 0.053–10.546 | 0.829 |
| Dyspnoea | 4.383 | 1.549–12.404 | 0.005 |
| Cough | 0.497 | 0.172–1.433 | 0.196 |
| Headache | 1.998 | 0.597–6.689 | 0.261 |
| Chest tightness | 1.436 | 0.525–3.924 | 0.481 |
| Fever | 0.983 | 0.350–2.766 | 0.974 |
| Diarrhoea | 0.436 | 0.147–1.295 | 0.135 |
| Loss of smell | 1.182 | 0.439–3.184 | 0.741 |
| Fatigue | 2.512 | 0.587–10.745 | 0.214 |
| Days after infection | 1.988 | 1.047–3.773 | 0.036 |

# 2. *Logistic Regression analysis of WPAI among male participants*

| **Variable** | **Odds Ratio** | **95% CI** | **p-value** |
| --- | --- | --- | --- |
| Intercept | 1.444 | 0.503–4.148 | 0.495 |
| Age (years) | 1.500 | 0.906–2.483 | 0.115 |
| BMI | 1.080 | 0.730–1.597 | 0.700 |
| Severe COVID-19 | 0.674 | 0.229–1.983 | 0.474 |
| COVID-19 ICU | 1.305 | 0.460–3.699 | 0.617 |
| Dyspnoea | 0.615 | 0.274–1.381 | 0.239 |
| Cough | 0.650 | 0.314–1.347 | 0.247 |
| Headache | 1.171 | 0.560–2.448 | 0.674 |
| Chest tightness | 1.821 | 0.860–3.857 | 0.118 |
| Fever | 2.484 | 1.109–5.567 | 0.027 |
| Diarrhoea | 2.327 | 1.050–5.157 | 0.038 |
| Loss of smell | 0.565 | 0.279–1.142 | 0.112 |
| Fatigue | 0.643 | 0.269–1.535 | 0.320 |
| Days after infection | 1.370 | 0.926–2.0 | 0.115 |
