# Supplementary material for: Sex-specific patterns of symptom burden, psychological distress, and work productivity impairment in post-acute sequelae of COVID-19: a 10-month dynamic cohort study
Source: BMC Pulm Med. 2026 Sep 19;26:418. doi: 10.1186/s12890-026-04741-x (PMC13589870; doi:10.1186/s12890-026-04741-x)
Supplement: Supplementary file 4 [file 12890_2026_4741_MOESM4_ESM.docx]

Supplementary Table 4. Sex-by-covariate interaction terms from the combined generalized linear mixed-effects model of work productivity impairment (WPAI-VAS ≥2), with Hom correction for multiple testing

| **Variable** | **OR** | **95% CI** | **p value** | **Holm-adjusted p value** |
| --- | --- | --- | --- | --- |
| Male sex:age | 2.08 | 0.97–4.44 | 0.059 | 0.649 |
| Male sex:bmi | 0.61 | 0.31–1.18 | 0.139 | 1.000 |
| Male sex:covid19 intensity severe | 1.24 | 0.19–8.24 | 0.820 | 1.000 |
| Male sex:covid19 intensity severe/ICU | 2.14 | 0.20–22.58 | 0.526 | 1.000 |
| Male sex:dyspnoea | 0.13 | 0.04–0.44 | 0.001 | 0.013 |
| Male sex:cough | 0.98 | 0.31–3.03 | 0.968 | 1.000 |
| Male sex:headache | 0.68 | 0.20–2.36 | 0.547 | 1.000 |
| Male sex:chest tighthness | 1.54 | 0.50–4.76 | 0.457 | 1.000 |
| Male sex:fever | 2.92 | 0.90–9.51 | 0.074 | 0.666 |
| Male sex:diarrhea | 5.47 | 1.61–18.61 | 0.006 | 0.072 |
| Male sex:loss of smell | 0.49 | 0.16–1.47 | 0.203 | 1.000 |
| Male sex:fatigue | 0.24 | 0.05–1.06 | 0.060 | 0.649 |
| Male sex:days since infection | 0.89 | 0.47–1.69 | 0.724 | 1.000 |
